# Syntheses, reactivity, and biological applications of coumarins

**DOI:** 10.3389/fchem.2024.1362992

**Published:** 2024-02-19

**Authors:** Andrea Citarella, Serena Vittorio, Christian Dank, Laura Ielo

**Affiliations:** ^1^ Dipartimento di Chimica, Università degli Studi di Milano, Milano, Italy; ^2^ Dipartimento di Scienze Farmaceutiche, Università degli Studi di Milano, Milano, Italy; ^3^ Institute of Organic Chemistry, University of Vienna, Vienna, Austria; ^4^ Department of Chemistry, University of Turin, Turin, Italy

**Keywords:** coumarins, synthesis, reactivity, biological applications, inhibitory activities

## Abstract

This comprehensive review, covering 2021–2023, explores the multifaceted chemical and pharmacological potential of coumarins, emphasizing their significance as versatile natural derivatives in medicinal chemistry. The synthesis and functionalization of coumarins have advanced with innovative strategies. This enabled the incorporation of diverse functional fragments or the construction of supplementary cyclic architectures, thereby the biological and physico-chemical properties of the compounds obtained were enhanced. The unique chemical structure of coumarine facilitates binding to various targets through hydrophobic interactions pi-stacking, hydrogen bonding, and dipole-dipole interactions. Therefore, this important scaffold exhibits promising applications in uncountable fields of medicinal chemistry (e.g., neurodegenerative diseases, cancer, inflammation).

## 1 Introduction

Coumarins represent one of the foremost privileged scaffolds, frequently existing in a huge variety of natural products and bioactive molecules (Stefanachi et al., 2018). The diverse array of biological characteristics (Srikrishna et al., 2018) has rendered this notable category of heterocyclic compounds appealing to medicinal chemists throughout the years. These properties encompass antioxidant (Stanchev et al., 2009), anticonvulsant (Keri et al., 2022), antitumor (Wu et al., 2020), anti-inflammatory (Grover and Jachak, 2015), and antimicrobial (Al-Majedy et al., 2017) activities. Several coumarin derivatives have been approved by FDA for clinical usage. These include anticoagulant drugs such as warfarin (Ansell et al., 2004), acenocoumarol (Cesar et al., 2004), dicoumarol (Duxbury and Poller, 2001) and phenprocoumon (Warkentin et al., 2022), trioxsalen (Sehgal, 1974) which is employed for the treatment of vitiligo, and esculin (Smith and Moodie, 1988) which is used in combination against hemorrhoids.

The ready availability and the low price of the starting materials required for synthesizing coumarins have enabled the development of a wide range of methodologies. Furthermore, the distinct reactivities associated with the C-3 and C-4 positions of the coumarin system have paved the way for selective modifications, introducing pertinent functional groups (such as fluorinated moieties) for medicinal chemistry scopes and facilitating the construction of cyclic systems. This broadens the potential of coumarins as a valuable starting point for the synthesis of more intricate chemical architectures. Classical methods used for the synthesis of coumarins include Knoevenagel (Bigi et al., 1999), Perkin (Johnson, 2004), Pechmann, (Pechmann, 1884; Yavari et al., 1998), Wittig (Yang et al., 2018), Claisen (Cairns et al., 1994), and Reformatsky (Shriner, 2004) reactions. The purpose of this discussion is to provide a comprehensive overview of the latest progress in the synthesis of 3-substituted, 4-substituted, and decorated or bicyclic coumarins, spanning from 2021 to the present. Moreover, a subsequent section presents the most relevant functionalization reactions of coumarins, for the selective introduction of diverse type of functional groups or for the construction of more complex cyclic derivatives. Finally, the various biological activities specific to coumarin derivatives are illustrated. The focus of this review was on the last 3 years, aiming to delineate the most significant advancements that have emerged since the publication of other reviews encompassing this field (Bouhaoui et al., 2021; Patil, 2022).

## 2 Syntheses

### 2.1 3-Substituted coumarin derivatives

**SCHEME 1:**
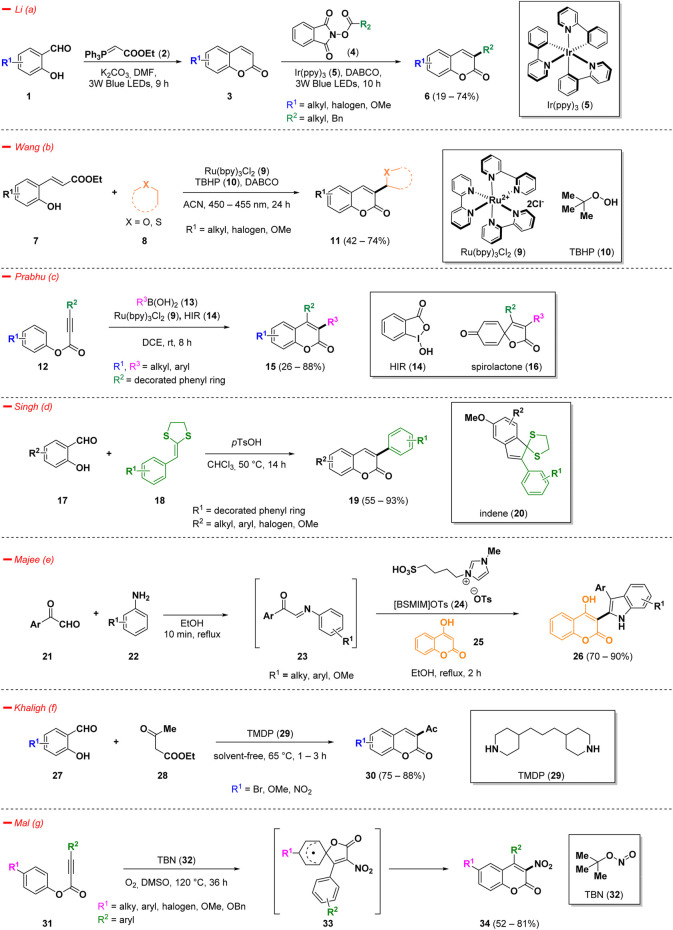
Synthesis of 3-substituted coumarins.

Increased interest on 3-substituted coumarins was observed, due to their biologically relevant applications in medicine and chemical biology (Vina et al., 2012; Stefanachi et al., 2018; Sokol et al., 2021). Therefore, the development of efficient and straightforward approaches for the synthesis of such scaffolds has garnered considerable attention (Xia et al., 2022). Recently, innovative strategies to access 3-alkyl, 3-heteroaryl, 3-acetyl, and 3-nitro coumarins have been developed, including green syntheses, photo- and metal-catalyzed reactions, and multi-component approaches, *inter alia* (Scheme 1).

Li *et al.* developed a structured one-pot method for the synthesis of 3-alkyl coumarins (**6**) using simple and cheap commercially available salicylaldehydes (**1**) (Kim et al., 2023). The reaction mechanism presumably involves a classical Wittig reaction to afford the coumarin ring (**3**), that *in situ* reacts with the proper alkyl donating reagents *N*-hydroxyphthalimide esters (**4**). The process smoothly occurs in presence of Ir(ppy)_3_ photocatalyst (**5**) under blue LEDs irradiation at room temperature and DABCO (1,4-diazabicyclo [2.2.2]octane), providing variously 3-substituted coumarin derivatives (**6**) based on the *N*-hydroxyphthalimide ester (**4**) chosen with a wide reaction scope (Scheme 1—*path a*). The selected catalytic system exhibited a good functional group tolerance and DMF resulted to be the most efficient solvent for the conversion.

In the context of photocatalytic strategies for the innovative synthesis of 3-functionalized coumarins, Wang et al. came across a direct and regioselective C(sp2)−C(sp3) coupling reaction of hydroxy cinnamic esters (**7**) with (thio)ethers (**8**) under the presence of Ru(bpy)_3_Cl_2_ (**9**) as a photocatalyst and TBHP (**10**, *tert*-butyl hydroperoxide) as an oxidant (Scheme 1—*path b*) (Zhang et al., 2021). Overall, the process could be described as a cascade reaction consisting of a first alkenylation of α-C(sp3)H bond of ethers/thioethers and a subsequent lactonization. Novelty of such a methodology could be found in its broad substrate scope, mild reaction conditions, and the possibility to realize a one-pot procedure from commercially available salicylaldehyde, under *in situ*-Wittig olefination.

Prabhu and collaborators have developed a visible-light-mediated functionalization of activated alkynes (**12**) for the synthesis of coumarin derivatives (**15**) (Scheme 1—*path c*) (Manna and Prabhu, 2023). Within radical-induced reactions, the notable reactivity of aryl alkynoates has garnered substantial interest, largely attributed to their ease of accessibility and distinctive ability to readily accept radicals. They exploited the capacity of boronic acids (**13**) to be effective alkyl sources in the presence of hypervalent iodine reagent **14** (HIR) and photocatalyst **9**. Using this methodology, it was possible to access several simple chain-alkylated 3-substituted coumarins (**15**) with broad functional group tolerance and good yields. Interestingly, the process demonstrated to be adaptable to the formation of a spirolactone compound (**16**) instead of coumarin whenever alkynoate starting material bearing a *p*-methoxy substituent was used.

In the context of 3-aryl substituted coumarins, an interesting advance has been found in the work of Singh and co-workers (Scheme 1—*path d*) (Arora et al., 2022) The authors described a simple, high yielding and metal-free Brønsted acid-catalyzed methodology to afford 3-aryl coumarins (**19**) starting from commercially available salicylaldehydes (**17**). The best condition to perform the process was the employment of *p*-toluenesulfonic acid (*p*TsOH) as catalyst in refluxing chloroform, revealing excellent yields and broad substrate scope. Moreover, switching the starting material and modifying the reaction conditions, the methodology diverged to a facile synthesis method for indene derivatives (**20**).

Majee *et al*. reported a metal-free and eco-friendly procedure for an easy access of coumarin derivatives (**26**) functionalized in position 3 with an indole scaffold (Scheme 1—*path e*) (Samanta et al., 2022). The process proceeded via a tandem cyclization reaction of phenylglyoxal derivatives (**21**) and several substituted anilines (**22**) in a multicomponent approach in the presence of the Brønsted acidic ionic liquid [BSMIM]OTs (**24**, 1-butane sulfonic acid-3-methylimidazolium tosylate) as a green catalyst in refluxing ethanol. Considering the emerging importance of indole scaffold endowed with antitumor, antiviral and antifungal effects (Citarella et al., 2023; Jagadeesan and Karpagam, 2023; Kudličková et al., 2023), the authors decided to screen the library of 3-indole coumarins (**26**) *in silico* for their ability to bind key proteins in tumorigenesis revealing interesting outcomes, which was confirmed also by a preliminary bioactivity evaluation.

Khaligh and others conducted a green Knoevenagel condensation using 4,4′-trimethylenedipiperidine (**29**, TMDP) in solvent-free conditions affording 3-substituted coumarins (**30**) starting from variously decorated salicylaldehydes (**27**) (Scheme 1—*path f*) (Gorjian and Khaligh, 2022).

Aryl alkynoates (**31**) readily underwent cascade-type cyclization reactions with *tert*-butyl nitrite (**32**, TBN) to provide 3-nitro coumarins (**34**) in good yield, following a *5-exo-trig* pathway (Sau and Mal, 2021). The use of TBN (**32**) in nitration and nitrative cyclization reactions showed interesting eco-friendly and less-toxic advantages. The process discovered by Mal *et al.* is characterized by the formation of an intermediate spiro-compound (**33**) that after ester migration affords the desired 3-nitrocoumarins (**34**) (Scheme 1—*path g*). The presented robust methodology displayed high regioselectivity and good functional group tolerance. Control experiments conducted in the presence of the radical scavenger TEMPO (4-hydroxy-2,2,6,6-tetramethylpiperidin-1-oxyl) demonstrated that the reaction followed a radical pathway. Moreover, a conventional nitro-group reduction was conducted on a selected example and this approach represents an easy process to access biologically relevant 3-aminocoumarins.

### 2.2 4-Substituted coumarin derivatives

**SCHEME 2:**
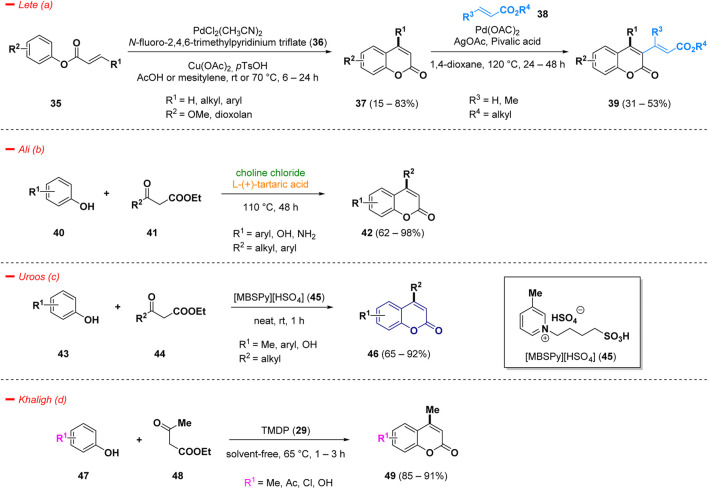
Syntheses of 4-substituted coumarins.

Examples of C-H functionalizations for the synthesis of diversified 4-substituted coumarins (**37**) were reported by Lete *et al*. and consisted in a Pd(II)-catalyzed direct C-H alkenylation (Fujiwara-Moritani reaction) (Ortiz-de-Elguea et al., 2021). The substrate scope analyzed by the author highlighted the facility to afford 4-substituted coumarins (**37**), bearing several types of aliphatic and (hetero)aromatic fragments in good yields, in acetic acid or mesitylene, the presence of copper as additive, and *N*-fluoro-2,4,6-trimethylpyridinium triflate (**36**) as oxidant. The obtained 4-functionalized skeleton (**37**) can be further modified *via* C3 intermolecular alkenylation to easily afford highly substituted coumarins (**39**) (Scheme 2— *path a*).

The acid-catalyzed Pechmann condensation is the classic and easiest method to access 4-functionalized coumarins and, in the most simple case, involves the reaction of substituted phenols and β-ketoesters/acids (Lončarić et al., 2020). Many of the reported procedures require the use of stoichiometric amounts of costly catalysts, producing of acidic wastes without any possibility of recycling, with dangerous environmental impacts. Therefore, the search for greener procedure for the synthesis of substituted coumarins represents a challenging task and, in this context, the use of deep eutectic solvents (DES), acting simultaneously as solvents and catalysts, represents a valuable way to achieve this goal. Ali and collaborator carried out a Pechmann condensation under green conditions, via the use of choline chloride and l-(+)-tartaric acid (1:2) at 110°C to achieve 4-functionalized coumarin derivatives (**42**) (Scheme 2—*path b*) (Rather and Ali, 2022).

Pechmann reaction was also investigated using an eco-friendly doubly Brønsted acidic task specific ionic liquid [MBSPy][HSO_4_] (**45**, 1-butylsulfonic-3-methylpyridinium hydrogen sulfate) as catalyst. Under a solvent-free process at room temperature it was possible to obtain substituted coumarin derivatives (**46**) in good yields, starting from phenols (**43**) and beta-ketoesters (**44**) (Scheme 2—*path c*) (Uroos et al., 2022). Moreover, the ionic liquid catalyst **45** could be reusable in accordance with green chemistry principles. The synthesized compounds were additionally assessed for their antifungal properties against *Macrophomina phaseolina*, a fungus that impacts over 500 plant species globally and lacks any specific commercially available fungicide, unrevealing novel potential applications for the mentioned scaffold (Marquez et al., 2021).

Another interesting green approach of the Pechmann reaction was discovered by Khaligh and others. They implemented the use of TMDP (**29**) as safe and greener catalyst for a facile synthesis of coumarin derivatives (**49**), functionalized in position 4 with a methyl group (Scheme 2—*path d*) (Gorjian and Khaligh, 2022)

### 2.3 Coumarins functionalized on the phenyl ring; polycyclic, and dihydrocoumarin derivatives

**SCHEME 3:**
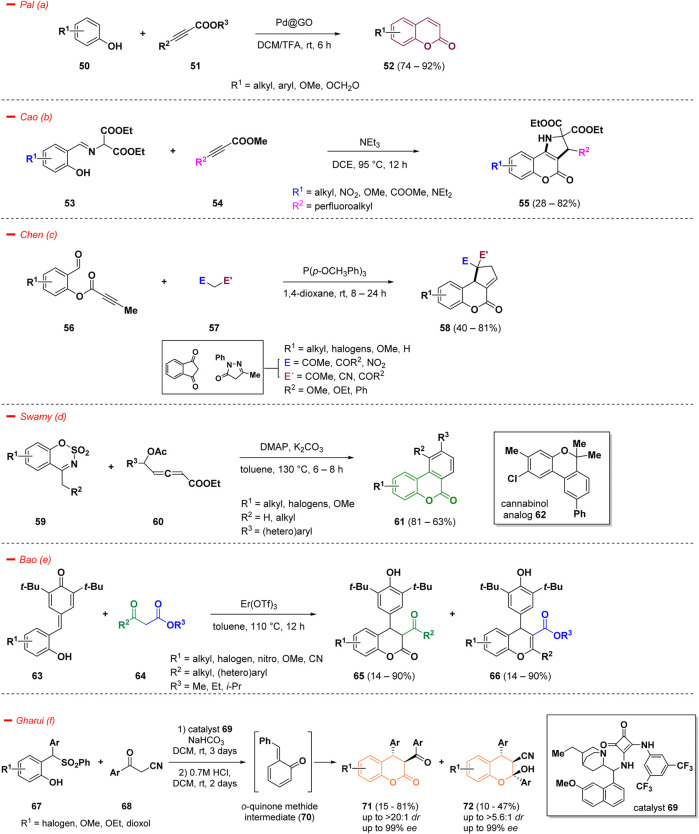
Synthesis of decorated coumarins.

An effective strategy to access coumarins decorated in the phenyl ring relies on C–H functionalization. However, this approach holds several limitations, especially concerning the use of non-recyclable homogenous catalytic systems. Pal *et al*. developed a stereo- and regio-selective C–H bond functionalization strategy to access in high yields decorated biologically relevant coumarins (**52**) starting from different substituted phenols (**50**) and alkynes (**51**), catalyzed by palladium nanoparticles supported on graphite oxide (Pd@GO) at room temperature (Kyndiah et al., 2023). The methodology showed improved catalytic efficiency and a good substrate scope with a low loading of the catalyst (Scheme 3— *path a*).

The recognition of heterocyclic fused rings has grown significantly, attributed to the improved pharmacological properties they offer, especially in nitrogen-containing architectures (Dank and Ielo, 2023). In particular, pyrrolidine-fused coumarins have been considered attractive targets in drug design, thanks to the biochemical relevance of both combined scaffolds (Ren et al., 2021). In this context, Cao and collaborators reported an efficient strategy for the synthesis of pyrrolidine-fused coumarins with perfluorinated side chains (**55**), utilizing several imines derived from aromatic aldehydes (**53**) and methyl β-perfluoroalkylpropiolates (**54**) as starting materials (Ren et al., 2021). The reaction comprises a tandem [3 + 2] cycloaddition and a subsequent intramolecular transesterification which afforded in a single step operation the fluoroalkylated final compounds (**55**) (Scheme 3—*path b*). The driving force of the entire process is represented by the strong electron withdrawing effect exerted by the perfluoroalkyl functionality. Moreover, the formation of such hyperfluorinated constructs can often ameliorate specific chemical-physical characteristics such as lipophilicity, binding selectivity, and metabolic stability, leading to a consistent improvement from the medicinal point of view (Swallow, 2015).

Regarding the development of efficient synthetic approaches to construct polycyclic ring systems, an interesting work from Chen and others introduced a novel phosphine-catalyzed, one-pot domino strategy for the annulation of 2-formylphenyl alkynoates (**56**) with activated methylene compounds (**57**) for the construction of several cyclopentene-fused dihydrocoumarins (**58**) (Chen et al., 2021). The authors intuitively designed an intramolecular cyclization strategy based on the reactivity of alkynoates (**56**) on the way to structurally diversified coumarins (**58**). Specifically, the process smoothly merged the reactivity of substituted 2-formylphenyl butynoates (**56**) and different 1,3-dicarbonyl compounds (**57**) in a tandem Knoevenagel condensation/[3 + 2] annulation leading to the target molecules under phosphine catalysis at room temperature in good yields (Scheme 3—*path c*).

A straightforward strategy for the synthesis of benzocoumarins (**61**) has been published by Swamy and co-workers (Chauhan et al., 2023). The authors highlighted a one-pot procedure taking advantage of the reactivity of cyclic sulfamidate imines (**59**) towards δ-acetoxy allenoates (**60**), herein investigated as a 5C-synthon for the construction of a π-extended coumarin skeleton under the simple catalysis of DMAP (Scheme 3—*path d*). The mechanism proceeds through a domino reaction, featuring sequential benzannulation and lactonization, to give the target benzocoumarins (**61**) in high yields. Moreover, the synthetic utility of the process was demonstrated by the conversion of one derivative into a cannabinol analogue (**62**).

4-Aryl-3,4-dihydrocoumarin and 4-aryl-4*H*-chromene are important structural derivatives of coumarins endowed with improved biological and pharmacological activities. In the work of Bao and others, a straightforward cyclization of *para*-quinone methide derivatives (**63**) with 1,3-dicarbonyls (**64**) was highlighted for the first time and the proposed strategy allowed the formation of a series of versatile 4-aryl-3,4-dihydrocoumarins (**65**) and 4-aryl-4*H*-chromenes (**66**) (Scheme 3—*path e*) (Bao et al., 2023). The reaction proceeded under the catalysis of Er(OTf)_3_ and represents an interesting approach for an easy access to structurally diversified coumarins and chromenes. The divergent approach was realized modulating the starting materials and maintaining the reaction conditions: the use of malonates afforded the exclusive synthesis of 4-aryl-3,4-dihydrocoumarins (**65**), while switching to beta-diketones provided exclusively the chromene derivatives (**66**).

Considering the importance of 3,4-dihydrocoumarins and tetrasubstituted chromans, another interesting approach to access such functionalized scaffolds was reported by Gharui *et al*. following an *in situ* generation of *o*-quinone methide intermediate (**70**) from sulfones (**67**) and a subsequent addition of aromatic α-cyanoketones (**68**). The methodology gave access to 3,4-dihydrocoumarins (**71**) and tetrasubstituted chromans (**72**) in high enantio- and diastereo-selectivities (Scheme 3—*path f*) (Gharui et al., 2021). The process proceeds under organocatalysis in good yields, however with prolonged reaction times.

## 3 Reactivity of coumarins

Structurally diverse coumarin derivatives have been synthesized by organic and medicinal chemists (Bouhaoui et al., 2021). The following discussion concerns the reactivity of the coumarin core, which was mainly investigated at the level of C-3 and C-4 of the pyranone ring. The following section is divided into a) general reactivity of coumarins, involving mainly the reactivity of C-3 whenever C-4 is unsubstituted, b) reactivity of 3-substituted coumarins, c) reactivity of 4-substituted coumarins, and d) reactivity of 3,4-disubstituted coumarins.

### 3.1 General reactivity of coumarins

**SCHEME 4:**
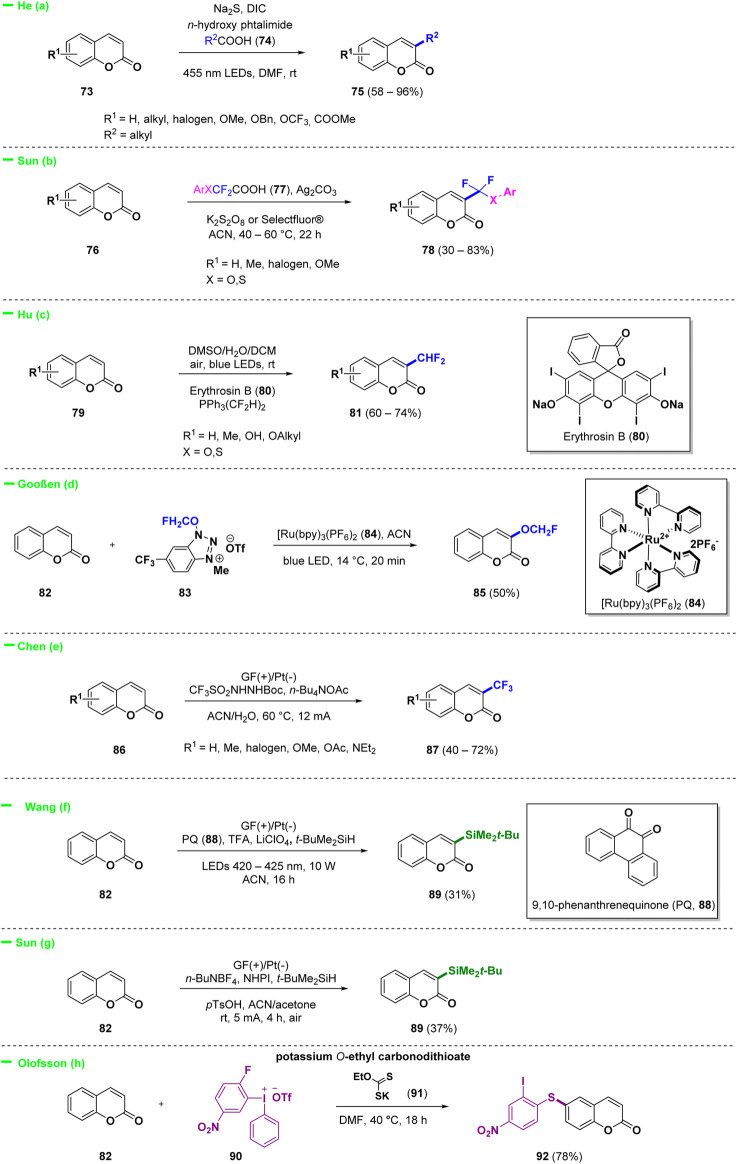
General reactivity of coumarins.

Recently, C-3 modification of coumarins was the most investigated approach to access a variety of interesting derivatives. A general scheme for the reactivity of C-3 substituted coumarins obtained under various conditions is reported below and describes the introduction of alkyl, silyl, CF_3_, CHF_2_, and OCH_2_F groups to afford biologically relevant scaffolds (Scheme 4). 3-Alkylated coumarins (**75**) were successfully synthesized by He and collaborators using a simple and practical electron donor-acceptor photochemical strategy (Scheme 4—*path a*) (Song et al., 2023). The protocol involved carboxylic acids (**74**) as starting materials, *in situ* activated by NHPI (*N*-hydroxyphthalimide) using Na_2_S as catalyst. Radical and photochemical approaches were also investigated *en route* to the introduction of fluorinated functionalities in position 3 of the coumarin system. Difluoromethylation today represents a straightforward manner to tune physico-chemical properties of pharmaceuticals and several research groups attempted to selectively incorporate this relevant functional group into bioactive compounds (Swallow, 2015; Miele et al., 2019; Citarella et al., 2022). Sun and collaborators realized a simple silver-catalyzed oxidative decarboxylation of arylthiodifluoroacetic acids or aryloxydifluoroacetics (**77**) for the selective C-3 functionalization of coumarins (**76**) with a difluoromethyl group (Scheme 4—*path b*) (Sun et al., 2022). Hu *et al*. proposed a selective photoredox catalysis-induced direct C-3 difluoromethylation of coumarins (**79**) by using bis(difluoromethyl) pentacoordinate phosphorane (PPh_3_(CF_2_H)_2_) and Erythrosin B (**80**) (Scheme 4—*path c*) (Song et al., 2023). Among fluorinated derivatives, also monofluoromethylation and trifluoromethylation recently gained a considerable attention among medicinal chemists. Gooßen and others proposed a photocatalytic C-3 functionalization of coumarin (**82**) under Ru(II) catalysis in the presence of 1-(OCH_2_F)-3-Me-6-(CF_3_)benzotriazolium triflate (**83**) as source of monofluoromethyl units (Scheme 4—*path d*) (Bertoli et al., 2023). On the other hand Chen and collaborators described an efficient eco-friendly electrochemical trifluoromethylation of C-3 position of coumarins (**86**), catalyst free, using CF_3_SO_2_NHNHBoc as the CF_3_ source (Scheme 4—*path e*) (Cen et al., 2023). Other interesting electrochemical approaches were described for the selective introduction of silyl group at C-3 position. Wang and others proposed an organoelectrophotocatalytic strategy for C-3 silylation of coumarin **82** using 9,10-phenanthrenequinone (**88**, PQ) both as an organocatalyst and as a hydrogen atom transfer (HAT) reagent (Wan et al., 2023) (Scheme 4—*path f*). A Minisci-type reaction under electrochemical conditions was discovered by Sun *et al*. (Jiang et al., 2023) for the synthesis of several silylated heterocycles including coumarin **89**, employing NHPI as the hydrogen atom transfer (HAT) catalyst (Scheme 4—*path g*). Finally, selective modification of C-6 of the coumarin scaffold with carbonodithioate salt **91** was achieved in the work of Olofsson *et al.* by using iodonium salt **90** as reactive arylation and vinylation reagent (Scheme 4—*path h*) (Mondal et al., 2023).

### 3.2 Reactivity of 3-substituted coumarins

#### 3.2.1 3-Acyl and 3-aryl coumarins

**SCHEME 5:**
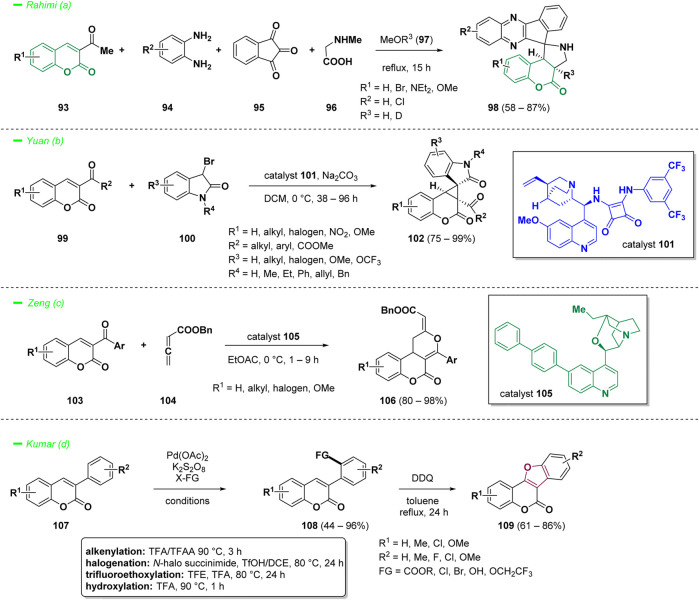
Reactivity of 3-acyl and 3-aryl coumarins.

The reactivity of 3-acetyl coumarins (**93**) in a multi-component reaction was investigated by Rahimi and others (Rahimi et al., 2023). The authors decided to merge the concept of multi-component reactions, an important approach today in medicinal chemistry for the synthesis of bioactive heterocyclic compounds, with a 1,3-dipolar cycloaddition strategy taking advantage of the reactivity of azomethine ylides with olefinic dipolarophiles. The methodology was employed to convert 3-acetyl coumarins (**93**) into novel chromeno [3,4-*c*]spiropyrrolidine-indenoquinoxalines (**98**), in a four-component 1,3-dipolar cycloaddition reaction with 1,2-phenylenediamines (**94**), ninhydrin (**95**), and sarcosine (**96**) (Scheme 5—*path a*). The best solvent for the process was found to be refluxing MeOH and in reaction times of 15 h, a wide panel of final compounds (**98**) were afforded with high regio- and stereoselectivity in moderate yields.

Another fascinating reactivity of 3-acyl or 3-aroyl coumarins (**100**) was discovered by Yuan and co-workers. The authors provided a valuable easy strategy for the synthesis of spirooxindole-cyclopropa [*c*]coumarins (**103**), merging two important pharmacophores such as cyclopropa [c]coumarins, an important member of the group of coumarin derivatives that include a cyclopropane unit, and spirooxindole derivatives, endowed with interesting biological applications (Scheme 5—*path b*) (Yuan et al., 2021). The reaction proceeds *via* a cyclopropanation reaction of the 3-acylcoumarin scaffold (**100**) with 3-halooxindole (**101**) catalyzed by a squaramide-based organocatalyst (**102**) through a [2 + 1] Michael/intramolecular cyclization. The methodology was optimized for 3-benzoyl coumarins, and the best reaction conditions were observed whenever the process was conducted using the squaramide catalyst (**102**) in DCM at 0 °C. The scope of the reaction included a variegated series of spirooxindole-cyclopropa [*c*]coumarin compounds (**103**) bearing three continuous stereocenters, including two vicinal quaternary carbon stereocenters, obtained with high yields.

Zeng *et al.* reported the synthesis of dihydrocoumarin-fused dihydropyranones (**106**) via a tertiary amine (**105**) catalyzed [4 + 2] cyclization of 3-aroylcoumarines (**103**) with benzyl 2,3-butadienoate (**104**) (Scheme 5—*path c*) (Li et al., 2021). Pyran moieties have been incorporated into numerous bioactive compounds, so that the synthesis of such structures attracted considerable interest during the last years. The scope of the reaction was explored by synthesizing a series of chiral dihydrocoumarin-fused dihydropyranones (**106**) using 6’-(4-biphenyl)-β-iso-cinchonine as catalyst (**105**) with optimal yields.

The work of Kumar and others offered a captivating example of *ortho* C−H bond activation for the synthesis of functionalized coumarins (**108**) that employs the lactone ring as weak coordinating group to direct a selective modification of 3-arylcoumarins (**107**) (Shinde et al., 2021). The methodology benefits from the cooperation of the catalyst Pd(OAc)_2_ and the oxidant K_2_S_2_O_8_ for the versatile alkenylation, halogenation, fluoroalkoxylation, and hydroxylation of variously decorated 3-arylcoumarins (**107**) (Scheme 5—*path d*). A broad scope of the reaction was examined, and a big variety of final products (**108**) were obtained with high yields. As an application, the so generated *o*-hydroxy derivatives were converted into bioactive coumestan (**109**) in a cyclization reaction mediated by DDQ (2,3-dichloro-5,6-dicyano-1,4-benzoquinone).

#### 3.2.2 3-Carboxy coumarins

**SCHEME 6:**
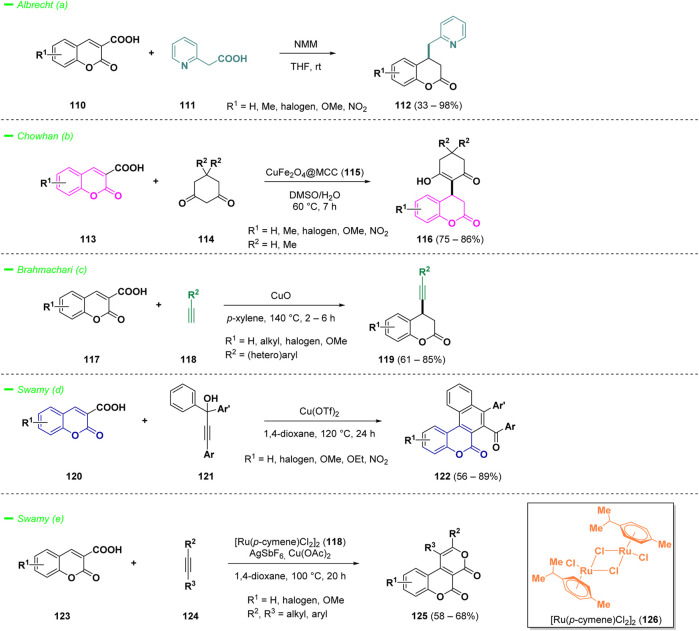
Reactivity of 3-carboxylic acid coumarins.

The synthesis of chromanones and bicyclic compounds primarily relies on harnessing the reactivity of 3-carboxylic acid coumarins, which is governed by their intrinsic decarboxylation potential. Albrecht *et al.* reported a doubly decarboxylative Michael type addition of pyridyl acetic acids (**111**) to coumarin 3-carboxylic acids (**110**), providing access to interesting 4-(pyridylmethyl)chroman-2-ones derivatives (**112**), bearing two bioactive heterocyclic scaffolds (Bojanowski and Albrecht, 2021). The process has been conducted under Brønsted base catalysis, specifically *N*-methyl morpholine (NMM), in THF at room temperature and many substituents were well-tolerated during the transformation, including electron-withdrawing groups, electron-donating groups, and bulky aromatic rings (Scheme 6—*path a*).

Merging the reactivity of Michael acceptors with decarboxylation of 3-COOH coumarins was also investigated in the eco-friendly approach published by Chowhan and others (Kumar et al., 2022). The authors synthesized a composite of copper ferrite oxide nanoparticles immobilized on microcrystalline cellulose (**115**, CuFe_2_O_4_@MCC) and studied its catalytic properties for the reactivity of 3-COOH coumarins (**113**) against cyclic 1,3-diketones (**114**) to construct 3,4-dihydrocoumarin frameworks (**116**) (Scheme 6—*path b*). The protocol demonstrated a wide substrate scope, affording the final products (**116**) with good yields. Additionally, the easily separable non-toxic catalyst enhances the efficiency of the work-up operation. An illustrative example on gram-scale of the mild process further underscored its applicability.

Brahmachari and co-workers reported a straightforward methodology for the efficient synthesis of functionalized 4-(aryl-/heteroaryl-ethynyl)chroman-2-ones (**119**) starting from coumarin-3-carboxylic acids (**117**) and terminal alkynes (**118**) (Bhowmick and Brahmachari, 2023). The formation of C (sp)−C (sp3) bonds was catalyzed by copper (II) oxide via a direct cross-coupling followed by decarboxylation. The protocol afforded a panel of 4-substituted coumarins (**119**) with high yields, without the use of any additional ligands or bases, showing a wide tolerance of diverse functional groups (Scheme 6—*path c*).

The reactivity of coumarins with carboxylic acid group in 3-position (**120**) with alkynes (**121**) was also investigated in the work of Swamy and others (Shankar and Swamy, 2023). The authors employed a decarboxylative annulation strategy for the construction of polycyclic heteroaromatic architectures such as naphtochromenones (**122**) (Scheme 6—*path d*). The process involved the reaction of coumarin-3-carboxylic acids (**120**) with *t*-Bu propargylic alcohols (**121**), following a Meyer-Schuster fashion *via* *in situ* generated α,β-unsaturated carbonyl compounds. The decarboxylation process, mediated by copper (II) catalysis, afforded a panel of novel naphtochromenones (**122**) with good yields. The same researchers reported also that ruthenium (II)-catalyst (**126**) could afford oxidative [4 + 2] annulation of coumarin-3-carboxylic acids (**123**) with alkynes (**124**) via the C-H activation to provide novel coumarin-fused pyranones (**125**) (Scheme 6—*path e*). (Shankar and Kumara Swamy, 2022)

#### 3.2.3 3-Nitro, 3-cyano, 3-acetamido, and *N*-methoxy-3-carboxamide coumarins

**SCHEME 7:**
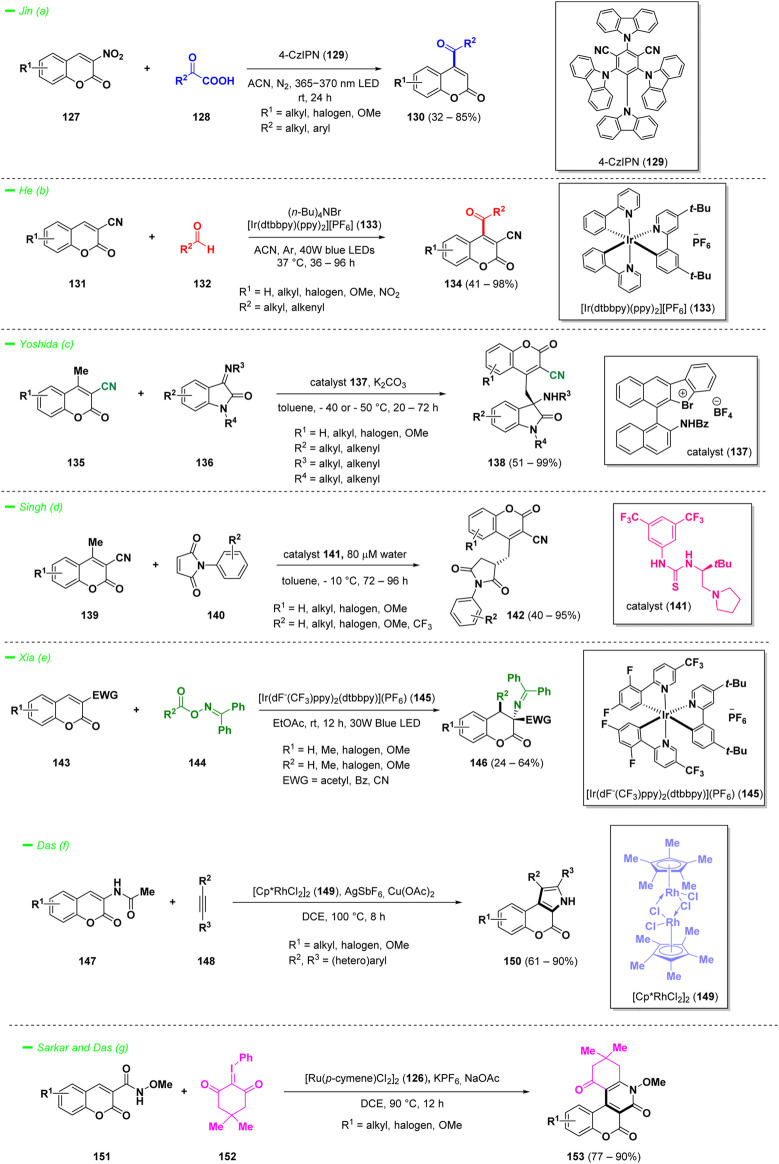
Reactivity of 3-nitro, 3-cyano, 3-acetamido, and *N*-methoxy-3-carboxamide coumarins.

3-Nitrocoumarins represent an intriguing class of compounds, and their versatility in organic synthesis stems from the ability to produce diverse derivatives of coumarins, particularly those with 4-acyl substitutions. Jin *et al.* discovered a novel green approach for the synthesis of C-4-acylated coumarins (**130**) starting from 3-nitrocoumarins (**127**) in the presence of α-keto acids (**128**) (Sun et al., 2023). The protocol demonstrated to efficiently work under mild conditions using photocatalysis, mediated by 4-CzIPN (**129**, 1,2,3,5-tetrakis (carbazol-9-yl)-4,6-dicyanobenzene) when the light source was a 365–370 nm LED (Scheme 7—*path a*). The main advantages of this methodology are the good yields and the wide tolerance of functional groups, moreover the process demonstrated to be oxidant-free and scalable.

Another straightforward example of C-4 acylation of 3-functionalized coumarins is represented by the visible light-induced cross-dehydrocoupling observed for 3-cyanocoumarin derivatives (**131**) during the reaction with aldehydes (**132**), discovered by He and others (Qian et al., 2023). The process took advantage of the inexpensive reagent (*n*-Bu)_4_NBr and the photocatalyst [Ir(ppy)_2_(dtbbpy)][PF_6_] (**133**) to functionalize the C-4 position of the coumarin with a large panel of acyl substituents derived from aliphatic and α,β-unsaturated aliphatic aldehydes (**132**), in good to excellent yields (Scheme 7—*path b*).

Among 3-nitrile substituted coumarins, the derivatives bearing a supplementary methyl group in position 4 are endowed with different types of vinylogous reactivity. Yoshida *et al.* in 2021 proposed an enantioselective Mannich-type reaction of 3-cyano-4-methyl coumarins (**135**) with iminoisatins (**136**) under the catalysis of a chiral bromonium salt (**137**) (Scheme 7—*path c*) (Yoshida et al., 2021). The same vinylogous-type reactivity was explored by Singh and collaborators studying the reactivity of similar 3-cyano-4-methyl coumarins (**139**) towards maleimides (**140**), as acceptors (Scheme 7—*path d*) (Singh et al., 2022). The process represented the first non-covalent organocatalytic enantioselective vinylogous Michael-type addition of 3-cyano-4-methylcoumarins (**139**) with maleimides (**140**) and demonstrated to be versatile affording a panel of final products (**142**) with yields up to 95%.

It is worth to mention another photocatalytic approach employed for the synthesis of variously substituted dihydrocoumarins (**146**) starting from coumarins substituted with an EWG at position 3 (**143**, Scheme 7—*path e*). The protocol reported by Xia and collaborators employed 3-CN, 3-acetyl, or 3-Bz substituted coumarins (as **143**) that were subsequently transformed into 4-amino dihydrocoumarins (**146**) via an alkylamination reaction at room temperature in EtOAc using [Ir(dF-(CF_3_)ppy)_2_(dtbbpy)](PF_6_) (**145**) as the photosensitizer (Jiang Y. S. et al., 2023).

Transition-metal-catalyzed annulation reactions of coumarin derivatives are important tools for the construction of coumarin-fused polycyclic heteroaromatic frameworks. Specifically, Das *et al.* had reported a valid two-step protocol to construct pi-extended *N*-heterocycles involving Rh(III)-catalyzed C–H activation starting from 3-acetamidocoumarins (**147**) and internal alkynes (**148**), whereas the acetyl group is involved as a traceless directing functional group to synthesize pyrrolo-coumarin complex heterocycles (**150**) (Scheme 7—*path f*) (Das and Das, 2022). The same research group reported the formation of privileged pi-extended coumarin-fused pyridone scaffolds (**153**), starting from 3-*N*-methoxy carboxamide coumarin compounds (**151**) in a [4 + 2] annulation reaction (Scheme 7—*path g*). In this case, a ruthenium (II) catalyst (**126**) has been employed for the process with optimal reaction yields (Sarkar et al., 2023).

### 3.3 Reactivity of 4-substituted and 3,4-disubstituted coumarins

Many biologically active pharmaceutics endowed with therapeutic effects, such as warfarin, dicumarol, coumafuryl, contain a 4-hydroxy coumarin core scaffold (Jung and Park, 2009). Moreover, tricyclic frameworks such as *trans*-2,3-dihydrofuro [3,2-*c*]coumarins (DHFCs) acquired enhanced interest during the last years as therapeutics and could be considered complex derivatives of 4-hydroxy coumarins, easily accessible from the latter through various reactions (Jang et al., 2012). Therefore, great efforts have been devoted to the construction of such heterocyclic architecture and lately the most elegant approaches have found to be multicomponent reactions and metal-catalyzed cycloadditions.

**SCHEME 8:**
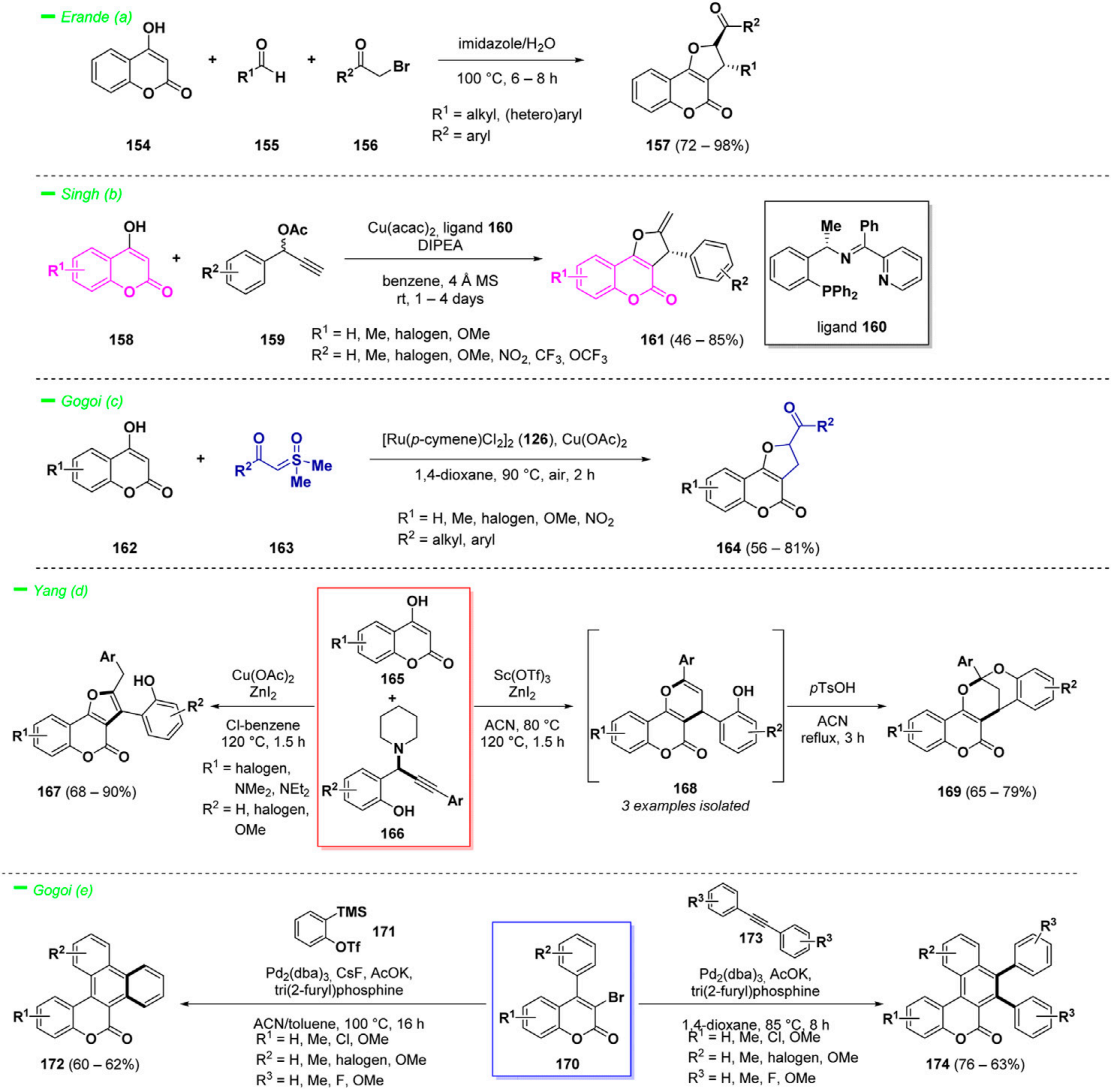
Reactivity of 4-hydroxy coumarins and 3,4-disubstituted coumarins.

Erande *et al.* reported an eco-friendly and efficient one-pot multi-component reaction to access such complex structures (Mali et al., 2022). The process involved the one pot reaction of 4-hydroxy coumarin derivatives (**154**) with aldehydes (**155**) and α-halo ketones (**156**) in a green solvent mixture of water and imidazole, affording the target compounds (**157**) in high yields. In this case the solvent mixture acted also as catalyst (Scheme 8—*path a*).

Another interesting approach for the synthesis of chiral dihydrofurocumarins (**161**) was proposed by Singh and others (Rohilla et al., 2023). This protocol took advantage of the reactivity of 4-hydroxy coumarins (**158**) as C,O-bis-nucleophiles in [3 + 2] cycloaddition reactions with propargylic esters (**159**) under copper catalysis. The proposed strategy led to the synthesis of optically active dihydrofuro [3,2-*c*]coumarin analogues (**161**) in moderate to good yields and high enantioselectivities (Scheme 8—*path b*).

Another example with focus on metal-catalyzed synthesis of dihydrocoumarins relies on the reactivity of 3-hydroxy coumarins (**162**) for the preparation of dihydrofuran-fused compounds (**164**) was reported by Gogoi *et al.* (Phukon et al., 2023) The key point of the transformation is the three-component annulation reaction of hydroxycoumarins (**162**) with sulfoxonium ylides (**163**) mediated by the 1,4-dioxane acting simultaneously as methylene source and solvent, under ruthenium (II) catalysis (Scheme 8—*path c*).

Yang and collaborators described a versatile synthesis of complex furano [3,2-*c*]coumarins (**167**) and pyrano [3,2-*c*]coumarins (**168**) exploiting the reactivity of 3-hydroxy coumarins (**165**) in a Lewis acid-catalyzed cascade annulation with *o*-hydroxyphenyl propargyl amines (**166**) (Sorabad and Yang, 2023). The methodology demonstrated to be regioselective and afforded the target compounds in good yields. Moreover, the pyrano-derivatives (**168**) could be easily converted into the more stable dioxabicyclic saturated heterocycles (**169**) *via* an acid-mediated cyclization (Scheme 8—*path d*).

π-Extended coumarins possess widespread applications in materials science, in particular they are endowed with photo-physical properties (Christie et al., 2008). Such complex polycyclic structures could be obtained starting from 3,4-disubstituted coumarins *via* annulation reactions following C−H activation strategies. Gogoi *et al.* proposed a palladium-catalyzed alkyne and aryne annulation protocol for the synthesis of a wide range of π-extended coumarin derivatives (**172**) in good yields with good functional groups tolerance (Hazarika et al., 2023). The process is driven by C−H activation and the formation of two new C−C bonds represented the key to build up the ring system (Scheme 8—*path e*). The starting material of the reaction contains a 3-bromo group and a 4-aryl substituent and, by switching the employed conditions, it was possible to obtain variously substituted π-extended coumarins (**174**).

## 4 Biological applications of coumarin derivatives

### 4.1 Neurodegenerative diseases

#### 4.1.1 Anti-Alzheimer

**FIGURE 1:**
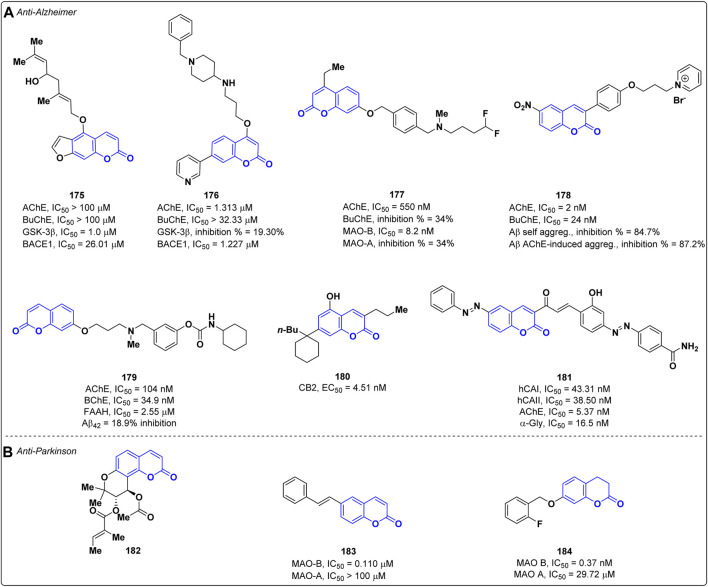
Coumarin-based compounds as anti-Alzheimer **(A)** and anti-Parkinson **(B)** agents.

Alzheimer’s disease (AD) is the most common type of dementia in elderly age, characterized by the progressive loss of cognitive functions. Despite the physio-pathological mechanisms responsible for AD have been not fully clarified, some key factors related to the neurodegeneration process have been identified, such as the loss of cholinergic neurons (cholinergic hypothesis), accumulation of Aβ amyloid fibrils (amyloid hypothesis) and *τ*-protein, oxidative stress and neuroinflammation (Breijyeh and Karaman, 2020). The therapies currently available for the cure of AD are mainly addressed to reduce the symptoms and, therefore, the search for more effective treatments represents an active research area. In recent years, multi-target directed ligands (MTDLs) have been envisaged as valuable strategy to develop new therapeutic agents for the cure of AD, and coumarin represents an appealing scaffold to address this task. In 2022, Zhao and co-workers adopted this approach by designing new coumarin derivatives that were tested against multiple targets relevant for AD, such as acetylcholinesterase (AChE), butyrylcholinesterase (BuChE), glycogen synthase kinase-3 beta (GSK-3β) and Beta-secretase 1 (BACE1) (Liu et al., 2022). AChE is an enzyme involved in the degradation of the neurotransmitter acetylcholine (ACh) whose inhibition increases the level of ACh prolonging its effects (Rees and Brimijoin, 2003). BuChE is an enzyme implicated in the hydrolysis of ACh and other choline derivatives, and it is mainly distributed in the peripheral nervous system. Most of the ChE inhibitors (ChEIs) employed in the therapy of AD blocks both AChE and BuChE; however, experimental studies highlighted that compounds selectively targeting AChE have a higher therapeutic index as BuChE inhibition can cause adverse effects to the peripheral nervous system (Li et al., 2016). On the other hand, GSK-3β is a kinase implicated in the phosphorylation and accumulation of τ-protein (Zhou et al., 2022) while BACE1 plays a pivotal role in the formation of Aβ fibrils (Hampel et al., 2021). To achieve the multi-target activity, the authors exploited the natural furanocoumarin notopterol **175**, known to inhibit both BACE1 (IC_50_ = 26.01 μM) and GSK-3β (IC_50_ = 1.00 μM), as lead compound. Among the synthesized derivatives, compound **176** (Figure 1A) displayed the highest affinity towards a) AChE (IC_50_ = 1.313 μM), with a selectivity index over BuChE (IC_50_ = 32.33 μM) of 24.623, and b) BACE1 (IC_50_ = 1.227 μM), thus showing better potencies than the lead compound notopterol on both targets. However, most of the designed derivatives exhibited a poor inhibition against GSK-3β without any improvement in respect to the lead, notopterol **175**. Kinetic studies highlighted that compound **176** acts as competitive inhibitor of AChE. Interestingly, derivative **176** proved to be able to cross the blood-brain barrier (BBB) in the parallel artificial membrane permeation assay for BBB (PAMPA-BBB) and to be safe at doses up to 1,000 mg/kg as demonstrated by in *in vivo* toxicity studies performed on mice (Liu et al., 2022). In the same year, Pisani *et al.* developed a new series of coumarins endowed with multi-target activity against AChE and monoamine oxidase B (MAO-B) (Rullo et al., 2022). The latter is an enzyme implicated in the oxidation of monoamine neurotransmitters in the brain and its activity increases in AD patients (Schedin-Weiss et al., 2017). The best inhibitory profile on both targets was yielded by derivative **177** (Figure 1A) which showed IC_50_ values of 0.550 μM and 0.0082 μM against AChE and MAO-B, respectively. In addition, compound **177** revealed to be selective for MAO-B over MAO-A, displaying a selectivity index superior to 1,250. Preliminary ADME investigations highlighted that the dual inhibitor **177** possesses a balanced lipophilic/hydrophilic profile, a high permeation through both the intestinal epithelial barrier and the BBB, and a high metabolic stability. According to kinetic studies, compound **177** is a competitive inhibitor of MAO-B, while it displayed a mixed-mode inhibition on AChE. In addition, no significant cytotoxic effects were observed for **177** in SH-SY5Y and HepG2 cell lines and a neuroprotective effect against both Aβ_1−42_ and H_2_O_2_ induced neuronal damage was exerted (Rullo et al., 2022). Khoobi *et al.* exploited the capability of pyridinium salt to interact with the catalytic anionic site of AChE developing a new class of coumarin derivatives cross-linked with pyridinium salt (Babaei et al., 2022). The most active compound **178** was able to inhibit both AChE and BuChE with IC_50_ values of 2.0 nM and 24.0 nM (Figure 1A), respectively, showing higher potency than donepezil (IC_50_ = 14.0 nM on AChE, IC_50_ = 2750 nM on BuChE; Structure not shown) used as reference. Moreover, derivative **178** was able to reduce the neuronal damage induced by H_2_O_2_ in PC12 cells and by Aβ_1−42_ in SH-SY5Y cells. It decreased both the Aβ self- (84.7% inhibition) and AChE-induced (87.2% inhibition) aggregation at 100 μM being more effective than the reference drug donepezil (30.8% inhibition on self- Aβ and 71.9% on AChE-induced aggregation).

In recent times, a newfound understanding of the relationship between the endocannabinoid system (ECS) and neuroprotection has emerged. The available evidence suggests that ECS signaling is implicated in the regulation of cognitive processes and plays a role in the pathophysiology of Alzheimer’s disease (AD). For this reason, pharmacotherapy targeting ECS could represent a valuable contribution, opening a new perspective for the development of active agents with multitarget potential. Rampa *et al.* reported a series of coumarin-based carbamic and amide derivatives as multipotent compounds acting on cholinergic system and ECS-related targets (Montanari et al., 2021). Their activity was evaluated on AChE and BChE, on fatty acid amide hydrolase (FAAH), and as cannabinoid receptor (CB1 and CB2) ligands. Moreover, their ability to reduce the Aβ_42_ self-aggregation was assessed. The most interesting profile was obtained for compound **179**, showing IC_50_ values of 104 nM on AChE, 34.9 nM on BuChE, 2.55 µM on FAAH, and only 18.9% of inhibition on Aβ_42_ (Figure 1A). Even if a significant activity of these compound against the CB1/CB2 receptors was not observed, this can be a starting point for further developments. Due to the involvement of ECS in numerous essential physiological and pathological processes, Bräse and co-workers evaluated the activity of different modified coumarins as cannabinoid receptor ligands (Mohr et al., 2021). The most active compound, **180**, showed a CB2 selective agonistic profile (*K*i = 6.5 nM, EC_50_ = 4.51 nM, see Figure 1A).

A multitarget approach was also chosen by Onar *et al.* for the design of new AD drugs (Çelik Onar et al., 2023). Twelve coumarin-chalcone derivatives were synthesized, and their biological activity was evaluated against AChE, human carbonic anhydrases (hCAs) I and II, and α–glycosidase (α-Gly). Derivative **181** showed promising results with IC_50_ values of 43.31 nM on hCA I, 38.50 nM on hCAII, 5.37 nM on AChE, and 16.5 nM on α-Gly, higher in comparison to the reference standards (Figure 1A). All the synthesized compounds showed acceptable physicochemical and pharmacokinetic properties.

#### 4.1.2 Anti-Parkinson

Parkinson’s disease (PD) is a progressive neurological disorder that mainly affects movement, causing tremors, stiffness, and difficulty with coordination and balance. The condition is characterized by the degeneration of dopamine-producing neurons in the brain, leading to a shortage of dopamine, a neurotransmitter essential for smooth and controlled muscle movements (Vittorio et al., 2020). The hallmark of PD consists of the presence of neuronal inclusions, called Lewi bodies, composed by phosphorylated and misfolded α-synuclein (α-syn) (Gitto et al., 2022). The inhibition of α-syn aggregation represents a promising disease-modifying strategy to halt or slow PD-related neurodegeneration (De Luca et al., 2022). In 2022, Kim and others reported the prevention of α-synuclein aggregation activity and the neuroprotective effect of the synthetic coumarin derivative PCiv (**182**) (Figure 1B) (Kim et al., 2022). This compound was able to inhibit α-syn aggregation *in vitro* and mitigated PFF-induced α-synucleinopathy in primary cortical neuron cultures. Preclinical investigations in a PD animal model revealed that PCiv (**182**) prevented the motor dysfunctions in the treated PD mouse model. *In vivo* studies confirmed the ability of PCiv (**182**) to permeate the BBB, despite a low bioavailability was observed in rats.

Another common strategy adopted in PD therapy is the use of selective inhibitors of MAO-B which is implicated in dopamine catabolism. In 2022, Matos and co-workers identified trans-6-styrylcoumarin **183** (Figure 1B) as selective inhibitor of human MAO-B with an IC_50_ values of 0.110 μM and a selectivity index over MAO-A (IC_50_ > 100 μM) greater than 900 (Mellado et al., 2022).

In 2023, Zhang and co-workers designed and synthesized novel 3,4-dihydrocoumarins as potent and selective MAO-B inhibitors (Liu et al., 2021). The best derivative is **184** with an IC_50_ value of 0.37 nM (reference iproniazid, IC_50_ = 7.69 nM) with a high selectivity towards MAO B (SI*>*270, MAO A IC_50_ = 29.72 µM) (Figure 1B). **184** acts as a competitive reversible inhibitor, effectively mitigating motor deficits in the MPTP-induced Parkinson’s disease model.

### 4.2 Antimicrobial

#### 4.2.1 Antibacterial derivatives

**FIGURE 2:**
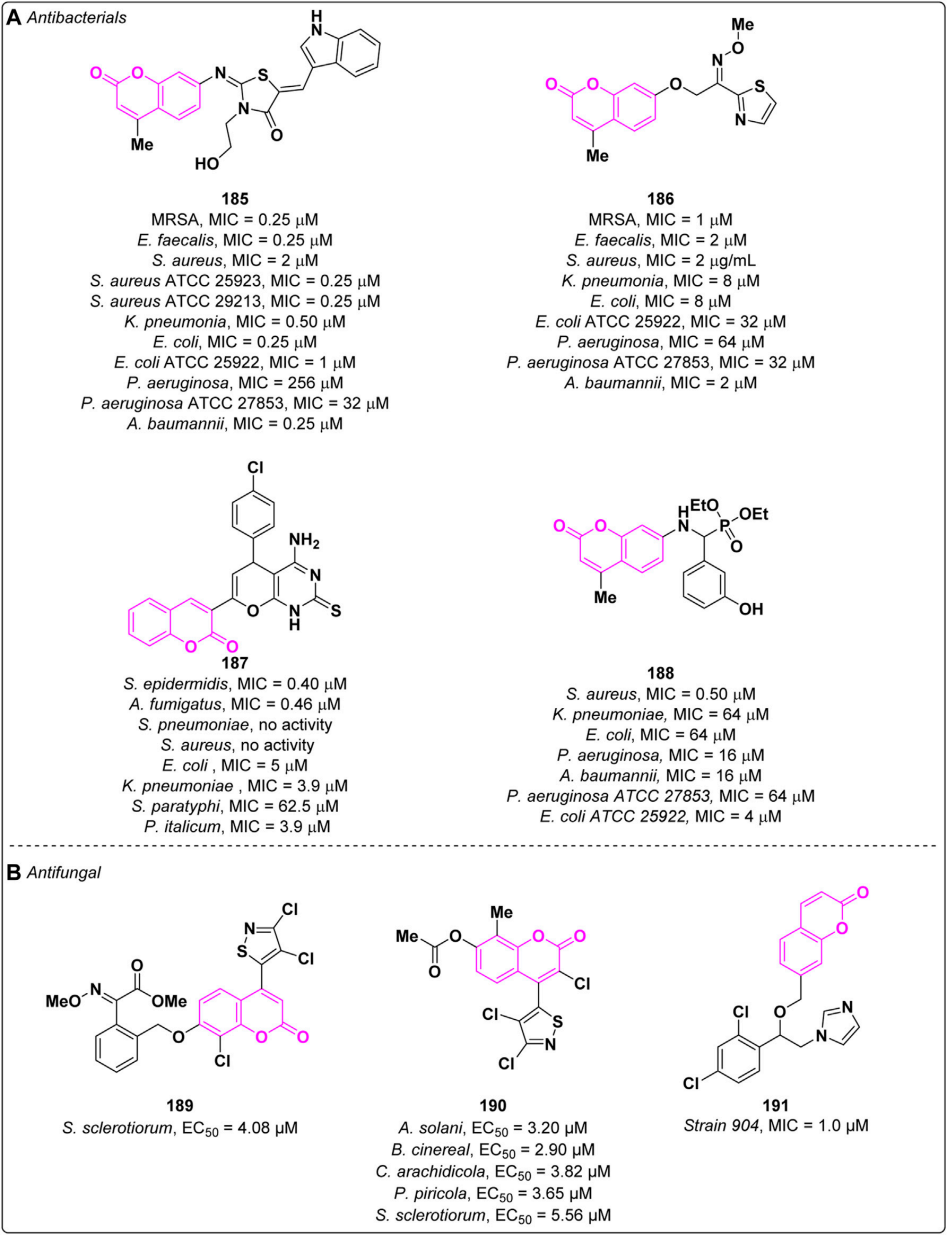
Antibacterial **(A)** and antifungal **(B)** activities of coumarin-based compounds.

Multidrug-resistant (MDR) bacterial infections represent a global emergency leading to the increase of the mortality rate due to the inefficacy of currently used antibiotics for the treatment of common infections (Serpi et al., 2023). In the last decade, several efforts have been made towards the discovery of broad spectrum antibacterials (Douglas et al., 2023). In this context, the coumarin moiety emerged as a promising scaffold for the design of potential antibacterial agents. The planar nature associated with the bicyclic ring facilitates interaction with vital biomacromolecules, such as DNA, making it an attractive choice for the development of intercalary agents. In 2022, Zhou and collaborators described the antibacterial activity of new thiazolidinone-conjugated coumarins which were tested against a panel of both Gram-positive and Gram-negative bacteria (Yang et al., 2022b). The most promising compound **185** displayed excellent activities on the tested bacteria, except for *P. aeruginosa* and *P. aeruginosa* ATCC 27853, at low concentrations (MICs = 0.25—2 μM) (Figure 2A). Additional experiments demonstrated that derivative **185** possesses the ability to disrupt the integrity of bacterial membranes and effectively reduces the formation of bacterial biofilms, without any significant cytotoxicity in mammalian cells. Compound **185** is less prone to drug resistance in comparison to the reference norfloxacin and does not show hemolytic activity. According to experimental and *in silico* studies, derivative **185** intercalates into DNA base pairs and interacts with DNA gyrase B, hampering its function. In the same year, the same research group also reported a new series of coumarin thiazoles endowed with antibacterial activity (Yang et al., 2022a). Among the tested derivatives, compound **186** (Figure 2A) exhibited a strong inhibition on methicillin-resistant *Staphylococcus aureus* (MRSA) showing a MIC value of 1 μM thus being more potent than norfloxacin and ciprofloxacin on the same strain (MICs = 8 μM). In addition, derivative **186** displayed a broad *spectrum* being able to inhibit different bacterial strains exhibiting good to moderate activity (MICs = 2—64 μM). Derivative **186** displayed no haemolytic effect along with the ability to eradicate MRSA biofilm. Moreover, it was also able to induce membrane damages leading to the leakage of intracellular material, to promote intracellular oxidative stress and to interact with DNA. Further studies highlighted a lower tendency of resistance of **186** against MRSA in comparison to norfloxacin.

In 2023, El-Kalyoubi et al. evaluated the antimicrobial activity of several nitrogen-containing coumarin derivatives (Fayed et al., 2022). Both Gram-positive (*S. Pneumoniae*, *S. Epidermidis*, *S. Aureus,* and *E. coli*) and Gram-negative (*K. Pneumoniae* and *S. Paratyphi*) bacteria were considered. Among the most promising derivatives **187** merits mention, which showed a MIC value of 0.40 µM on *Sclerotinia epidermidis*, in comparison to the standard (MIC = 15.6 µM) (Figure 2A). It also showed fungicidal activity against *A. fumigatus* (MIC = 0.46 µM; standard MIC = 15.6 µM).

Coumarin aminophosphonates (Koszelewski et al., 2023) are considered new antibacterial agents, able to combat bacterial resistance, as reported by Zhou and co-workers (Yang et al., 2023). Derivative **188** exhibited excellent inhibition potency against *S. aureus* (MIC = 0.5 µM; standard MIC = 16 µM) *in vitro* and showed considerable antibacterial potency *in vivo* (Figure 2A). It can eradicate the *S. aureus* biofilm, thus diminishing the development of *S. aureus* resistance. Furthermore, its combination with norfloxacin can enhance the antibacterial efficacy. Mechanistic explorations revealed that **188** was able to destroy the integrity of cell membrane, which resulted in the leakage of protein and metabolism inhibition.

#### 4.2.2 Antifungal derivatives

Similar to antibacterials, most of the antifungal agents currently employed present MDR along with the frequent occurrence of side effects. This prompted research to find more effective drugs (Prusty and Kumar, 2020).

A series of 21 novel 3,4-dichloroisothiazolocoumarin-containing strobilurins were rationally designed and synthesized by Fan and co-workers (Lv et al., 2022). Derivative **189** exhibited good antifungal activity against *Sclerotinia sclerotiorum* with a EC_50_ of 4.08 µM, (coumoxystrobin was used as reference, EC_50_ = 1.0 µM) (Figure 2B). The same research group reported in 2023 the fungicidal activity of 4-(3,4-dichloroisothiazole)-7-hydroxy coumarins ester derivatives (Song et al., 2023). Compound **190** displayed good efficacy against *Alternaria solani* (EC_50_ = 3.20 µM), *Botrytis cinereal* (EC_50_ = 2.90 µM), *Cercospora arachidicola* (EC_50_ = 3.82 µM), *Physalospora piricola* (EC_50_ = 3.65 µM), and *S. sclerotiorum* (EC_50_ = 5.56 µM), see Figure 2B.

In 2023, Gou et al. employed an alternative approach to avoid resistance (Yan et al., 2023). Specifically, certain coumarin derivatives endowed with antibiofilm activity were combined with CYP51 inhibitors to synthesize novel compounds with robust antifungal capabilities and reduced susceptibility to resistance. Compound **191** exhibited fungicidal effects against fluconazole-resistant *strain 904* (MIC = 1 µM) (Figure 2B). Most importantly, **191** showed to be potent as *in vivo* antifungal activity against pathogenic fungi and fluconazole-resistant strains was observed. Preliminary pharmacokinetic and toxicity tests demonstrated the drug-like properties of this compound.

#### 4.2.3 Antivirals

**FIGURE 3:**
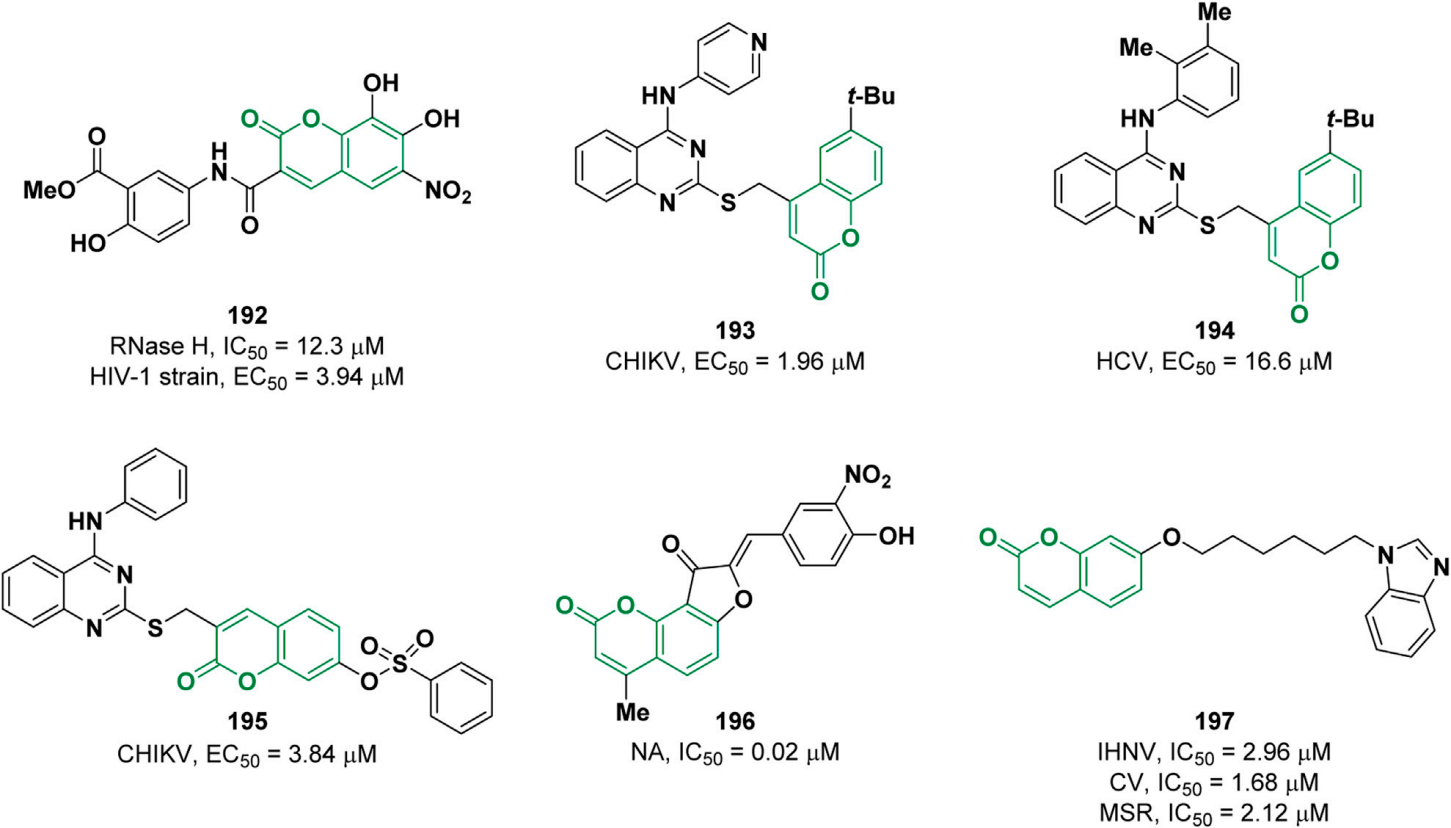
Coumarin derivatives possessing antiviral activities.

Viral infections constitute an important global health problem. For most of the pathogenic viruses like severe acute respiratory syndrome (SARS), Ebola, Zika, Chikungunya (CHIKV) no effective therapeutic treatments and/or vaccines are available. Therefore, there is an urgent need to find new and effective anti-viral drugs (Yoshida et al., 2021). Over the years, coumarin derivatives have been widely explored as promising antiviral agents (Li et al., 2022). In 2021, Zhan and co-workers discovered some coumarin derivatives as human immunodeficiency virus type 1 (HIV-1) inhibitors (Kang et al., 2021). After the screening, compound **192** was found to be the most active with an IC_50_ of 12.3 µM (DW-4, IC_50_ = 20.8 µM), in an enzymatic assay against the viral RNase H (Figure 3). **192** showed increased potency in comparison to the reference compound (DW-4, EC_50_ = 101 µM) against wild-type HIV-1 strain (EC_50_ = 3.94 µM) and retained activity against a panel of mutant strains.

In 2022, the antiviral activity of a series of quinazolin-4-amine -SCH_2_- coumarin conjugated compounds was determined by Neyts *et al.* against chikungunya (CHIKV) and hepatitis C (HCV) viruses (Hwu et al., 2022). Derivative **193** exhibited an EC_50_ value of 1.96 μM on CHIKV while derivative **194** an EC_50_ value of 16.6 μM on HCV (Figure 3). In the same year, the same research group reported the activity of a further series of functionalized quinazoline-coumarin hybrids carrying an arylsulfonate moiety against CHIKV (Hwu et al., 2022). Through a computational approach the authors designed compound **195** (Figure 3) which proved to be the most effective among the tested molecules with an EC_50_ value of 3.84 μM. The authors speculated that derivative **195** might interact with the nsP3 enzyme of CHIKV forming a covalent adduct with nucleophilic residues of the pocket through a Michael addition involving the coumarin moiety.

Pang and co-workers described a series of dihydrofurocoumarin derivatives as neuraminidase (NA) inhibitors, a promising target for the development of anti-influenza drugs (Zhong et al., 2021). The most potent inhibitor **196** possesses an IC_50_ value of 0.02 μM, lower in comparison to the reference oseltamivir carboxylate (IC_50_ = 0.04 μM) (Figure 3).

Chen et al. evaluated the activity of 35 new coumarin derivatives against infectious hematopoietic necrosis virus (IHNV) (Hu et al., 2021). The inhibitor with the best activity is **197** with an IC_50_ value of 2.96 µM. Furthermore, **197** showed IC_50_ values of 1.68 and 2.12 µM for two other rhabdoviruses, spring viremia of carp virus (CV) and *Micropterus salmoides* rhabdovirus (MSR), respectively (Figure 3). *In vivo* studies showed that **197** exhibited an anti-rhabdovirus effect in virus-infected fish by substantially enhancing the survival rate.

### 4.3 Anti-inflammatory

**FIGURE 4:**
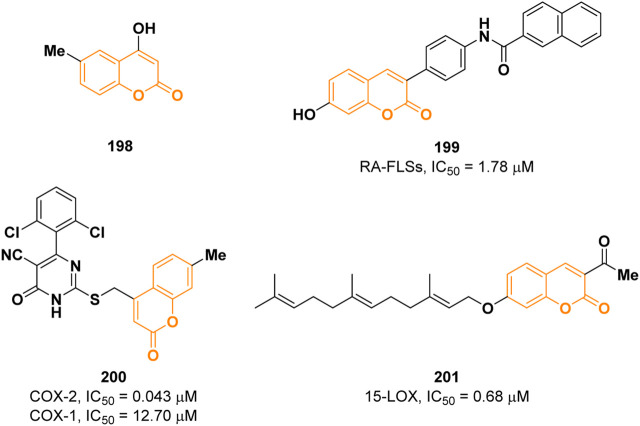
Coumarin derivatives with anti-inflammatory properties.

Inflammation can be defined as a complex response of the immune system triggered by harmful stimuli such as pathogens, damaged cells, irradiation, or toxic compounds (Chen et al., 2018). There are two discernible types of inflammation: acute and chronic. The former starts rapidly upon infections and last for a few days, while the latter is a slow and long-term process involved in some pathologies such as diabetes, cardiovascular disease, allergies, arthritis, and chronic obstructive pulmonary disease (Zotova et al., 2023). The inflammatory reaction is mediated by the release of several molecules including pro-inflammatory cytokines, nitric oxide (NO), prostaglandin E_2_ (PGE_2_), that are involved in different biological pathways regulating the inflammatory response (Matthay et al., 2003). Despite several anti-inflammatory agents are currently used in therapy, they possess different side effects; therefore, the development of safer anti-inflammatory drugs is still an attractive research field (Almasirad et al., 2022). In 2021, Kang and others described the anti-inflammatory activity of four coumarins among which the 4-hydroxy-6-methylcoumarin **198** (Figure 4) resulted to be the most active (Kang et al., 2021). Coumarin **198** was able to reduce the levels of the pro-inflammatory cytokines IL-1β, IL-6, TNFα, and PGE_2_ by 80.6%, 73.1%, 32.8%, and 53.2%, respectively, in LPS stimulated RAW 264.7 cells in a dose-dependent manner at a concentration of 500 μM. Western blot analysis confirmed the capability of **198** to downregulate the expression of iNOS and COX-2, two proteins implicated in the regulation of NO and PGE_2_ levels, respectively. In addition, it was demonstrated that **198** inhibits both MAPK and NF-kB signalling pathways.

Wang and co-workers reported a series of novel 3-(4-aminophenyl) coumarins as anti-inflammatory drugs for the treatment of rheumatoid arthritis (RA) (Miao et al., 2021). Preliminary results showed that compound **199** possesses the strongest inhibitory activity, among the tested compounds, on the proliferation of fibroid synovial cells (RA-FLSs, IC_50_ = 1.78 µM) compared to the reference methotrexate (IC_50_ = 5.0 µM), and it also has inhibitory effect on RA related cytokines IL-1, IL-6, and TNF-α (Figure 4). Mechanistic studies showed that **199** could inhibit the activation of NF-_*k*_B and MAPKs signal pathway. The anti-inflammatory activity was further determined *in vivo* in the rat joint inflammation model.

Non-steroidal anti-inflammatory drugs (NSAIDs) are among the most widely used medications to alleviate inflammation. They exhibit their effects *via* cyclooxygenase enzymes (COX) inhibition. COX enzymes exist in two distinct isoforms: COX-1 which is responsible for maintenance of physiological functions such as gastrointestinal integrity; COX-2 is responsible for proinflammatory conditions. Traditional NSAIDs with higher selectivity for COX-1 cause greater gastrointestinal bleeding, ulcer, and renal toxicity than those selectively targeting COX-2. Consequently, several studies led to the development of selective inhibitors of COX-2 isoform (coxibs) (Citarella et al., 2022). A new series of pyrimidine-5-carbonitrile-based coumarin derivatives was synthesized by Alfayomy et al. and their inhibitory activity was evaluated on both COX-1 and COX-2 (Alfayomy et al., 2021). Among them, derivative **200**, shown in Figure 4, showed the most promising potency with an IC_50_ value of 0.043 µM on COX-2 (reference celecoxib, IC_50_ = 0.045 µM) presenting a selectivity index of 295.35 (IC_50_ COX-1 = 12.70 µM). **200** displayed superior anti-inflammatory activity *in vivo* in comparison to celecoxib and during ulcerogenic liability testing, the compound was associated with mild lesions, comparable to celecoxib.

Concerning inflammations, lipoxygenases (LOXs) are well known to play an important role. They are nonheme iron-containing proteins that contribute to a new eicosanoid pathway by acting as biocatalysts in arachidonic acid’s peroxidation at positions 5, 8, 12, and 15 to the corresponding hydroperoxide derivatives. In this context, Seyedi and co-workers reported a novel array of geranyloxy and farnesyloxy 3-acetylcoumarins as potent soybean 15-lipoxygenase inhibitors (Zerangnasrabad et al., 2021). 7-Farnesyloxy-3-acetylcoumarin (**201**) was found to be the best inhibitor with an IC_50_ value of 0.68 µM (reference 4-MMPB, IC_50_ = 18 µM) (Figure 4). Docking studies revealed that the farnesyl moiety is well inserted in the hydrophobic cavity of the enzyme.

### 4.4 Anti-diabetes

**FIGURE 5:**
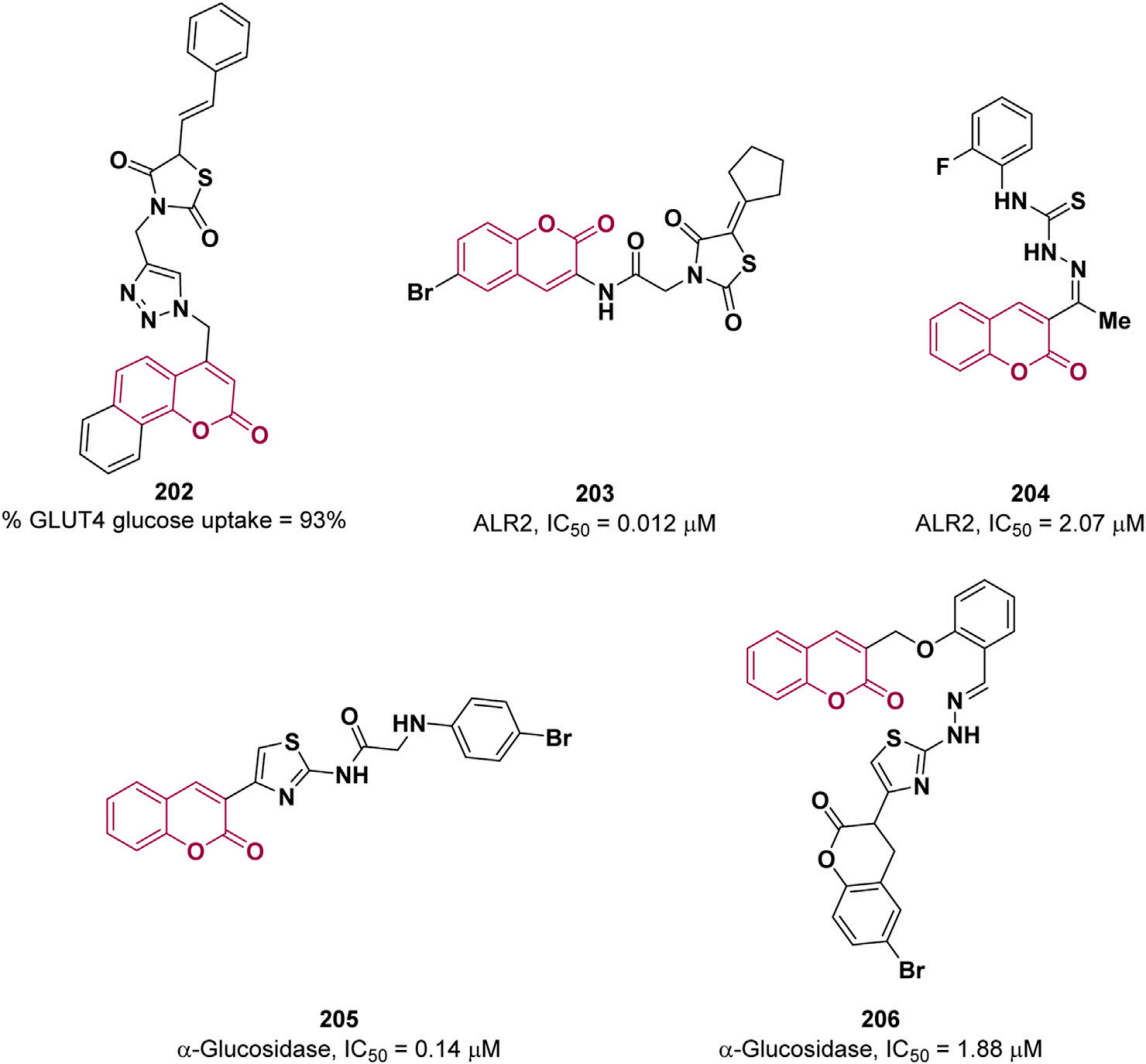
Coumarin derivatives having anti-diabetic potential.

Type-2 diabetes mellitus is a chronic metabolic disorder characterized by insulin resistance and impaired insulin secretion, associated with an enhancement of blood glucose levels. Lifestyle factors such as poor diet, sedentary behavior or obesity significantly contribute to its development. If left untreated, type-2 diabetes can result in serious complications, including cardiovascular diseases, kidney damage and brain dysfunctions (Munana, 1995). In skeletal muscle, accounting for the absorption of more than 80% of insulin-stimulated glucose, glucose uptake is mediated by protein carriers, namely, GLUT1 and GLUT4, whose function is impaired in T2DM. Therefore, the modulation of GLUT activity can be exploited for T2DM therapy. In 2023, Kamble et al. adopted a hybridization approach to design new anti-diabetic agents by combining three different pharmacophores: coumarin, 1,2,3-triazole, and thiazolidine-2,4-diones (Metre et al., 2023). The GLUT4 glucose uptake activity of the resulting compounds was evaluated on a yeast model leading to the identification of compound **202** (Figure 5) as the most effective with 93% glucose uptake at 200 μM, which is comparable to that of the reference pioglitazone (94%) at the same concentration. No significant cytotoxicity was detected by MTT assay for derivative **202**.

Coumarin and thiazolidinedione scaffolds were also employed by Pasala and co-workers to design new potential antidiabetics targeting aldose reductase-II (ALR2), an enzyme implicated in the conversion of glucose to sorbitol overactivated in diabetes (Kumar Pasala et al., 2021). Among the derivatives, the best activity was shown by **203** (IC_50_ = 0.012 μM) which proved to be selective towards ALR2 (selectivity index of 324.166), a forty-fold superiority over sorbinil (IC_50_ = 0.47 μM) (Figure 5). *In vivo* experiments suggested that **203** delays the progression of cataract in rats in a dose-dependent manner warranting its further development as potential agent to treat the diabetic secondary complications, especially cataract.

Iqbal et al. reported coumarin-thiosemicarbazone hybrids as ALR2 inhibitors (Imran et al., 2021). Compound **204** proved to be the most promising inhibitor with an IC_50_ = 2.07 μM (reference sorbinil, IC_50_ = 2.745 µM) and high selectivity, relative to ALR1 (Figure 5). The X-ray crystal structure of **204** in complex with ALR2 revealed the most important interactions and partially explain the strong binding affinity towards ALR2. A common strategy, used to reduce post prandial glycemia, consists of the inhibition of α-glucosidase, an enzyme involved in the digestion of carbohydrates, whose inhibition delayed the absorption of glucose. In an attempt to find novel, safe and effective α-glucosidase inhibitors, Vora et al., proposed coumarin linked thiazole derivatives as potential scaffold on the basis of their interactions with the active site of α-glucosidase studied *in silico* (Ichale et al., 2023). The most active compound, **205**, showed an IC_50_ value of 0.14 µM, in comparison to acarbose (IC_50_ = 6.32 µM), shown in Figure 5.

The coumarin based azomethine-clubbed thiazoles synthesis was documented by Al-Harrasi and co-workers in 2023 (Ul Ain et al., 2023). The authors evaluated the *in vitro* activity of the obtained compounds against α-glucosidase for the plausible treatment of diabetes mellitus (T2DM). The highest inhibition was observed for **206** with an IC_50_ value of 1.88 µM, in comparison to the reference acarbose (IC_50_ = 873.34 µM) (Figure 5). Docking studies were employed to predict the binding mode of the synthesized derivatives, revealing the significance of the interactions established by the azomethine moiety. This observation helps to explain the enhanced efficacy of the inhibitor.

### 4.5 Anticancers

The term “cancer” refers to a broad range of diseases characterized by an abnormal cell proliferation promoted by the mutations of genes implicated in the regulation of cell division and growth. These mutations can be induced by several factors such as irradiations, viruses, bacteria, smoking, and chemical compounds. Despite several progresses have been achieved in the anticancer therapy field, most cancers are still incurable. This prompted researchers to deeply study the cellular mechanisms involved in tumors, allowing the discovery of druggable targets that can be addressed for the development of novel therapeutic agents (Hassanpour and Dehghani, 2017).

**FIGURE 6:**
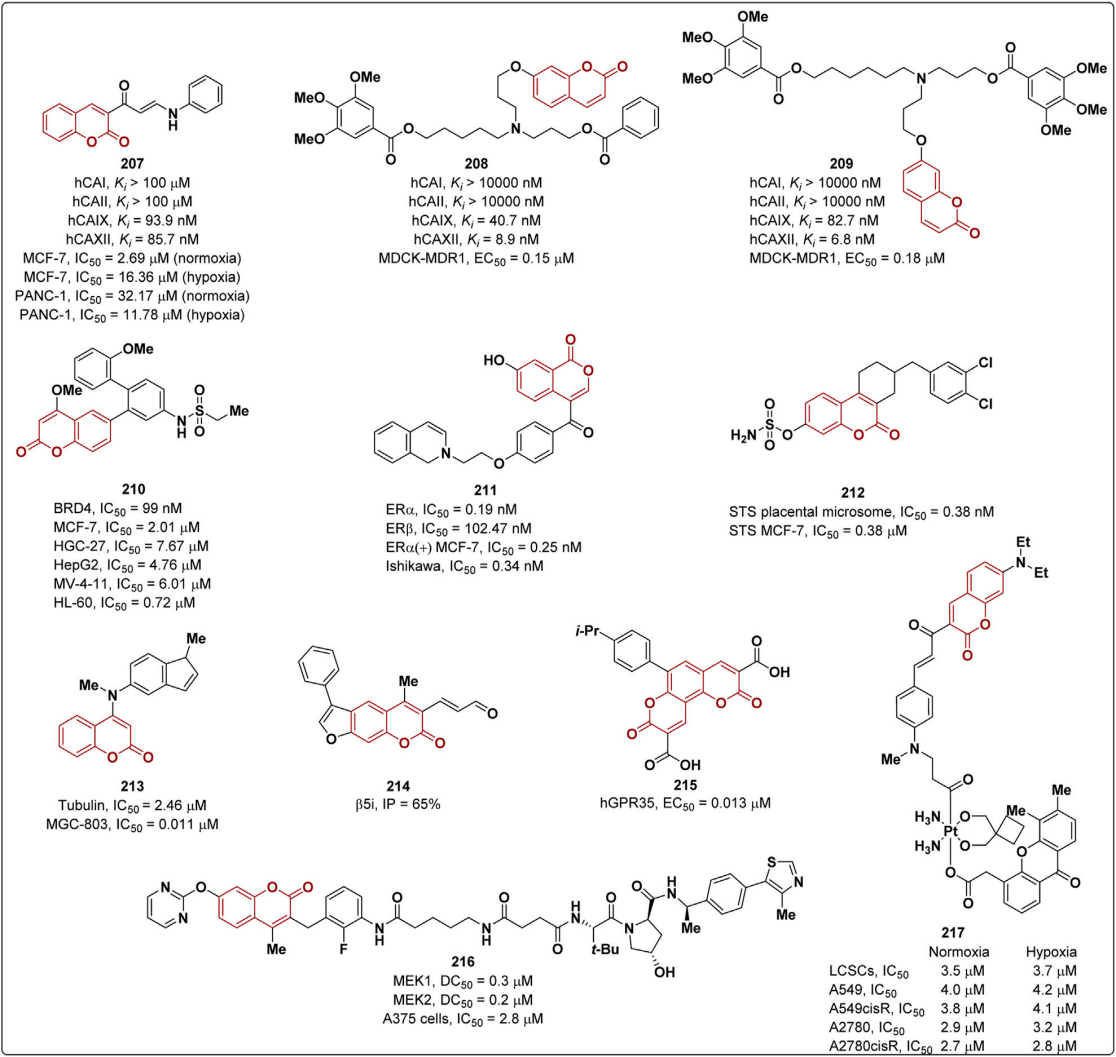
Coumarins with anticancer properties.

Coumarins showed anticancer activity targeting different proteins implicated in cancer-related pathways. Recent advances concerning the identification of coumarin-based compounds endowed with anticancer activity are reported in the following sections (Figure 6).

#### 4.5.1 Coumarins as inhibitors of carbonic anhydrase IX and XII

Human carbonic anhydrases (hCAs) are zinc containing enzymes catalyzing the reversible hydration of carbon dioxide to bicarbonate ions and protons. Among the 15 different isoforms of hCA identified so far, hCA IX and XII are implicated in tumor progression and have been widely recognized as pharmacological targets for anticancer therapy (Moi et al., 2022). In the last years, coumarins have emerged as selective hCA IX and XII inhibitors. Within this scenario, Eldehna *et al.* described the hCA inhibitory activity of novel coumarin-based aryl enaminone derivatives (Ibrahim et al., 2022). As results compound **207** (Figure 6) showed the highest selectivity towards the tumor associated isoforms hCA IX (*K*i = 93.9 nM) and hCA XII (*K*i = 85.7 nM) with selectivity ratios over the two ubiquitous isoforms hCA I and hCA II higher than 1,000, being more active than the reference compound acetazolamide (selectivity ratios between 0.5 and 43.9). The antiproliferative activity of **207** was evaluated on breast cancer MCF-7 and pancreatic cancer PANC-1 cell lines under both normoxic and hypoxic conditions. In the first case **207** displayed a more potent antiproliferative activity on MCF-7 cells (IC_50_ = 2.69 μM) than PANC-1 cells (IC_50_ = 32.17 μM). Under hypoxic conditions, a moderate inhibition was observed on both MCF-7 (IC_50_ = 16.36 μM) and PANC-1 cell lines (IC_50_ = 11.78 μM). Moreover, compound **207** delayed the cell cycle and induced apoptosis in MCF-7 cells.

A recent study revealed that hCA XII regulates the activity of P-glycoprotein (P-gp), a transporter protein associated with MDR involved in the active transport of chemotherapeutic drugs in the extracellular milieu reducing their cytotoxic effect. More specifically, the inhibition of hCA XII leads to a reduction of the intracellular pH which decreases Pgp activity (Kopecka et al., 2015). Therefore, the dual inhibition of hCA XII and Pg-p represents an appealing strategy to overcome Pg-p mediated MDR. Dei and others adopted a hybridization strategy to design novel hCA XII and Pg-p dual inhibitors (Braconi et al., 2022). In particular, the authors combined the *N,N*-bis(alkanol)amine diester moiety, which is known to interact with P-gp, with the coumarin scaffold. The best inhibitory profile was shown by compounds **208** and **209** (Figure 6) which inhibited hCA XII with *K*i values of 8.9 and 6.8 nM, respectively, and P-gp activity in MDCK transfected cells with EC_50_ values of 0.15 and 0.18 μM, respectively. Both compounds were able to restore doxorubicin antineoplastic activity in HT29/DOX and A549/DOX cells which overexpress both proteins thus revealing to be promising P-gp mediated MDR reversers.

#### 4.5.2 Coumarins as BRD4 inhibitors

BRD4 belongs to the bromodomain and extra-terminal (BET) protein family which comprises epigenetic proteins involved in the regulation of gene expression. In 2022, Cui et al. identified new BRD4 inhibitors bearing a coumarin scaffold (Cui et al., 2022). Among the synthesized derivatives, inhibitor **210** was identified as most promising anticancer agent. Compound **210** (Figure 6) inhibited BRD4 activity with an IC_50_ value of 99 nM and it was found to exert an antiproliferative activity in MCF-7 (IC_50_ = 2.01 μM), HGC-27 (IC_50_ = 7.67 μM), HepG2 (IC_50_ = 4.76 μM), MV-4-11 (IC_50_ = 6.01 μM) and HL-60 (IC_50_ = 0.72 μM) cell lines, without significantly affecting normal cells. In addition, the coumarin derivative **210** determined the arrest of cell cycle at G0/G1 phase and induced apoptosis in MCF-7 cells. Interestingly, compound **210** was also able to reduce the expression and transcription of c-Myc protein.

#### 4.5.3 Coumarins as antagonist of human estrogen receptor α

Human estrogen receptor α (ERα) is a nuclear transcription function whose activation by estrogens is responsible for an increment of cellular proliferation in breast cancer (Paterni et al., 2014). Therefore, the selective inhibition of ERα constitutes an approach for pharmacological intervention in breast cancer therapy. In 2022, Kurtanović et al. reported a new set of coumarin derivatives as selective ERα antagonists (Kurtanovic et al., 2022). The most potent compound (**211**, see Figure 6, IC_50_ = 0.19 nM) displayed not only a good selectivity over ERβ (IC_50_ = 102.47 nM) but also a higher inhibitory activity than the reference raloxifene (IC_50_ = 0.74 nM). Furthermore, derivative **211** exerted antiproliferative activity on ERα(+) MCF-7 cells (IC_50_ = 0.25 nM) and also on Ishikawa endometrial adenocarcinoma cell lines (IC_50_ = 0.34 nM) being more effective than raloxifene (IC_50_ of 0.89 and 0.94 nM on MCF-7 and Ishikawa cells, respectively). Compound **211** affected Raf-1/MAPK/ERK signal transduction pathway causing the arrest MCF-7 cell cycle at G_0_/G_1_ phase. The antitumoral activity of **211** was confirmed by *in vivo* experiments performed on a female Wistar rat breast cancer tumor model.

#### 4.5.4 Coumarins as steroid sulfatase inhibitors

Steroid sulfatase (STS) is an enzyme implicated in the hydrolysis of aryl and alkyl steroids sulfates thus playing a crucial role in the production of biologically active steroids. STS is overexpressed in breast cancer and its inhibition reduces estrogen formation, hindering tumor proliferation thus paving the way towards the development of anticancer agents (Foster, 2021). Within this context, Chang and others designed novel coumarin-7-*O*-sulfamate derivatives as STS inhibitors (Chang et al., 2022). The most promising compound **212** (Figure 6) disrupted STS activity from human placenta and MCF-7 cells with IC_50_ values of 0.38 and 0.38 μM, respectively, showing comparable potency to irosustat, which was used as reference and also has a coumarin core (IC_50_ of 0.20 and 0.16 μM, respectively). Prior studies pointed out that the sulfamate moiety covalently binds to STS and therefore irreversibly inhibits its function. Considering this, kinetic studies were conducted to evaluate the *K*i/*k*_inact_ ratio, which indicates the efficacy of covalent inhibition. Molecules with high *K*i/*k*_inact_ values might exert their biological activity at low doses showing a longer half-life. As result, derivative **212** provided a *K*i/*k*_inact_ value of 17.5 which is higher than that of irosustat (16.1). It is worth noting that compound **212** elicited antiproliferative effects on STS overexpressed cancer lines while being safe on normal cell lines.

#### 4.5.5 Coumarins as tubulin polymerization inhibitors

Microtubules, formed by α and β-tubulin heterodimers, are essential components of the cytoskeleton. Compounds affecting tubulin polymerization targeting the colchicine binding site exert a remarkable antitumor activity by interfering with tumor cell division (Lu et al., 2012). Within this context, Song and co-workers reported a new series of tubulin polymerization inhibitors obtained by combining the indole and coumarin moieties as both scaffolds proved to affect tubulin polymerization (Song et al., 2022). The most potent compound **213** (Figure 6) was able to inhibit tubulin polymerization by interacting with colchicine binding site with an IC_50_ value of 2.46 μM, showing a higher activity than colchicine (IC_50_ = 6.70 μM). Derivative **213** displayed a significant antiproliferative activity on gastric cancer cell line MGC-803 with an IC_50_ of 0.011 μM. Moreover, it promoted cell apoptosis, inhibited cell cycle at G2/M phase and cell migration in MGC-803 and HGC-27 gastric cancer cell lines. Finally, compound **213** exhibited antitumoral effects *in vivo*.

#### 4.5.6 Coumarins as proteasome inhibitors

The ubiquitin-proteasome system is responsible for the maintenance of protein homeostasis and the regulation of various cellular processes. Proteasome inhibition is a strategy employed in the anti-cancer therapy (Ielo et al., 2022; Ielo et al., 2023). Sosič and co-workers reported a series of coumarin derivatives as selective (immuno)proteasome inhibitors (Schiffrer et al., 2021). The most promising derivative resulted to be **214** with an inhibition percentage (IP) of 65% on the chymotrypsin-like (β5i) subunit (Figure 6).

#### 4.5.7 Coumarins as hGPR35 inhibitors

A series of coumarin-like diacid derivatives were designed and synthesized by Liang et al. as novel agonists of human G protein-coupled receptor 35 (hGPR35) which is implicated in a variety of pathologies including cancer (Wei et al., 2021). An EC_50_ value of 0.013 µM was determined for compound **215**, which proved to be one of the most active of the series (Figure 6).

#### 4.5.8 Coumarins as MEK1/2 inhibitors

The RAF/MEK/ERK pathway is a fundamental signal path associated with the proliferation, differentiation, and apoptosis of tumors. MEK1/2 is a key kinase target in the pathway and ERK1/2 is its main substrate. Even if several MEK1/2 inhibitors were reported, acquired resistance remains a significant problem. Xu and co-workers designed and synthesized a series of coumarin-based MEK1/2 PROTAC MEK1/2 degraders based on a coumarin derivative which was a potent non-diarylamine allosteric MEK1/2 inhibitor effective in human cancer cells (Wang et al., 2023). **216** is the most promising derivative showing a DC_50_ values of 0.3 and 0.2 μM in MEK1 and MEK2 degradation, respectively. Furthermore, it significantly inhibits the growth of A375 cells (IC_50_ = 2.8 μM) (Figure 6).

#### 4.5.9 Coumarins in light-driven cancer therapy

Light-driven cancer therapy including photodynamic therapy (PDT) represents an appealing strategy to cure tumors. This approach presents several advantages if compared to conventional chemotherapy, such as low invasiveness, the lack of cross-resistance, as well as spatially and temporally controllable activation. PDT usually relies on photosensitizers able to induce biomolecules damages by ROSs (Reactive Oxygen Species) production, thus requiring oxygen to exert the cytotoxic effects. However, tumoral cells are characterized by a hypoxic environment which limits the efficacy of PDT. To overcome this limitation, intracellular pH (pHi) homeostasis can be modulated to achieve anticancer effects. In 2023, Deng and collaborators described the development of a near-infrared (NIR) activated platinum (IV) complex **217** (Figure 6) carrying a coumarin-based photosensitizer as first example of Pt (IV) complex activatable in a two-photon excitation (TPE) manner, which allows a deeper IR tissue penetration (Deng et al., 2023). Complex **217,** localized in the endoplasmic reticulum, displayed low toxicity in the dark while exerting a significant antiproliferative effect on different cancer cell lines under both normoxic and hypoxic conditions (IC_50_ = 2.7–4.2 μM), shown in Figure 6 thus suggesting an oxygen-independent photocytotoxic effect. Indeed, **217** interfered with pHi and was able to trigger the immune system and reduce tumor growth and metastasis formation.

### 4.6 Miscellaneous

#### 4.6.1 Anti-leishmanial

**FIGURE 7:**
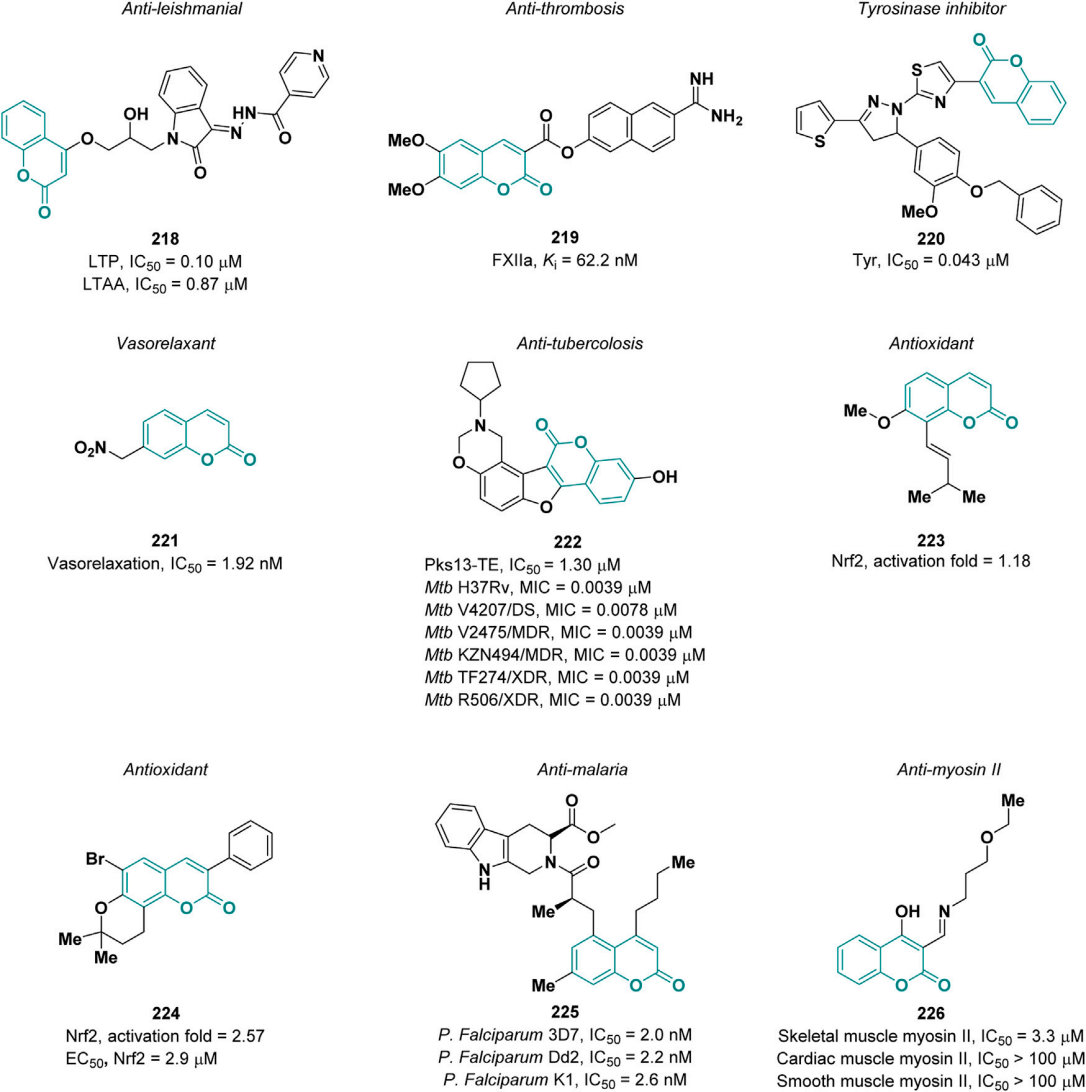
Coumarin derivatives endowed with miscellaneous pharmacological properties.

Leishmaniasis is one of the most common parasite infections worldwide and has restricted therapeutic options. Novel coumarin-isatin hybrids were synthesized by Naseer and co-workers in order to evaluate their activity as anti-leishmanial agents (Khatoon et al., 2021). Docking studies suggested which of the prepared derivatives installed profitable interactions with the target, leading to the identification, via *in vitro* assays, of the best derivative, **218**, with an IC_50_ value 0.10 μM and 0.87 μM against *L. tropica* promastigote (LTP) and axenic amastigote (LTAA) forms, respectively (references tartar emetic IC_50_ = 7.28 μM and Amphotericin B IC_50_ = 1.86 μM, respectively) (Figure 7).

#### 4.6.2 Anti-thrombosis

Pochet et al. reported coumarins as inhibitors of factor XIIa (FXIIa), a promising target for artificial surface-induced thrombosis and different inflammatory diseases (Davoine et al., 2023). By using a fragment-based drug discovery approach, they designed a new class of coumarin derivatives. The most potent compound **219** possesses a *K*i of 62.2 nM on FXIIa and it was tested in plasma to evaluate its stability and efficacy on coagulation assays (Figure 7). It showed a plasmatic half-life of 1.9 h and a good selectivity for the intrinsic coagulation pathway over the extrinsic one.

#### 4.6.3 Anti-tyrosinase

Tyrosinase (Tyr) is a key enzyme in the biosynthesis of melanin pigments (Mirabile et al., 2021). An excessive production of melanin can cause hyperpigmentation disorders such as melanoma (Vittorio et al., 2023). Kim and co-workers described a new series of thiophenyl-pyrazolylthiazole-coumarin hybrids as tyrosinase inhibitors (Nasab et al., 2023). The best activity was observed for derivative **220**, with an IC_50_ value of 0.043 µM (reference kojic acid, IC_50_ = 18.521 µM), which is a non-competitive inhibitor (Figure 7). It also demonstrated excellent antioxidant activity against DPPH and no cytotoxicity on B16F10 melanoma cells.

#### 4.6.4 Vasorelaxant

Coumarins can act as NO donor drugs thus exerting a vasorelaxant effect. In 2022, Toimil and collaborators reported a new series of nitrate-coumarins that were tested in contraction-relaxation studies on rat aorta precontracted with phenylephrine (Matos et al., 2022). The most active compound **221** (Figure 7) showed an IC_50_ value of 1.92 nM displaying superior potency than nitroglycerin (IC_50_ = 12.73 nM) and sodium nitroprusside (IC_50_ = 4.32 nM) used as reference.

#### 4.6.5 Anti-tuberculosis

Coumarin-based compounds exert anti-tubercular activity targeting the thioesterase (TE) domain of Pks13 enzyme implicated in the biosynthesis of mycolic acids which constitute the major components of *Mycobacterium tuberculosis* (*Mtb*) cell wall (North et al., 2014). Within this scenario, Yu and co-workers reported the design of a new series of coumarin derivatives endowed with anti-tubercular activity (Zhang et al., 2022). The most active derivative **222** (Figure 7) displayed a MIC value of 0.0039 μM proving to be more potent than the reference drugs isoniazid (MIC = 0.04 μM), rifampin (MIC = 0.125 μM) and ethambutol (MIC = 1 μM). Compound **222** inhibited Pks13-TE activity with an IC_50_ value of 1.30 μM and resulted to be effective also on clinical resistant strains of *Mtb*, namely, DS-TB (V4207), MDR-TB (KZN494 and V2475), and XDR-TB (TF274 and R506), showing MIC values between 0.0039 and 0.0078 μM (Figure 7). Interestingly, derivative **222** exhibited a good human microsomal stability and oral bioavailability in mice.

#### 4.6.6 Antioxidant

Coumarins can elicit antioxidant effects by acting as Nrf2 (nuclear factor erythroid 2-related factor 2) agonists. Indeed, the Keap1-Nfr2 pathway represents the main protective response to oxidative stress. In physiological conditions, Keap1 regulates the ubiquitination of Nrf2, while under oxidative stress conditions Keap1 dissociates from Nrf2 which in turn translocate into the nucleus promoting the transcription of cytoprotective genes (Buendia et al., 2016). In 2022, Ma and others described the Nrf2 agonistic activity of a new series of coumarin derivatives designed from osthole **223** (Figure 7), a naturally occurring coumarin able to increase the expression levels of Nrf2 (Huang et al., 2022). The most active compound **223** (Figure 7) showed an activation fold of 2.57 at a concentration of 20 μM, proving to be more effective than osthole (activation fold of 1.18). The EC_50_ value of **224** was measured on 293T cells yielding a value of 2.9 μM. Cellular thermal shift assay (CETSA) confirmed KEAP1 engagement by **224** in cellular environment.

#### 4.6.7 Anti-malaria

Coumarins were also investigated as therapeutics for the treatment of malaria which represents still today a global emergency due to the inefficacy of the currently employed chemotherapeutic agents against resistant *P. falciparum* strains. To find new and more effective antimalarial agents, Cho and collaborators designed a series of coumarins derivatives bearing a tetrahydro-β-carboline moiety (Cho et al., 2022). A phenotypic approach was employed to assess the ability of the synthesized derivatives to inhibit the malaria parasite growth. As result, compound **225** (Figure 7) was identified as the most potent antimalarial agents with an IC_50_ value of 2.0 nM against the wild-type *P. falciparum* strain 3D7, showing a higher activity than chloroquine (IC_50_ = 14 nM) used as reference. Moreover, **225** displayed a comparable inhibitory activity also against the chloroquine resistant strains Dd2 and K1 with IC_50_ values of 2.2 and 2.6 nM, respectively. Finally, derivative **225** decreased parasite growth in *P. berghei* ANKA-infected ICR mouse models, despite a short plasma half-life was observed.

#### 4.6.8 Anti-myosin II

The inhibition of muscle myosin is of interest to develop therapeutic agents for the treatment of hypercontractile states. On this ground, Bell and others designed novel 4-hydroxycoumarin imines as muscle myosin II inhibitors (Smith et al., 2023). Among the synthesized derivatives, compound **226** (Figure 7) showed the highest selectivity towards skeletal myosin (IC_50_ = 3.3 μM) over the cardiac and smooth muscle isoforms (IC_50_ > 100 μM). Interestingly, derivative **226** did not affect non-muscle myosin in cytokinesis assay and did not exhibit significant cytotoxicity.

## 5 Discussion

Natural compounds constitute an invaluable source of biologically active molecules. Coumarins are an excellent example of natural products presenting multiple pharmacological properties thus representing a privileged scaffold in medicinal chemistry.

Over the years, advances in synthetic organic chemistry went hand in hand with development of new strategies for the synthesis and functionalization of coumarins. Straightforward selective functionalization approaches permitted the incorporation into the coumarin system of several fragments, such as biologically relevant fluorinated moieties, alkyl/aryl substituents, or rigidified cyclic architectures, endowing the resulting scaffold with enhanced biological or physico-chemical properties. Relevant applications mostly occurred at the level of C-3 and C-4 positions of the coumarin scaffold, which constitute two important sites of functionalization, characterized by a unique type of reactivity. Considering the numerous applications of coumarins among chemical and biological sciences, there is still an urgent need for innovative strategies to expand the synthetic accessibility and the functionalization of such compelling scaffold.

From a medicinal chemistry point of view, the peculiar structure of coumarin, characterized by its planarity and lipophilicity, feature mostly impaired by the presence of a cyclic lactone moiety, enables the binding to diverse targets through hydrophobic, *pi*-stacking, hydrogen bonding, and dipole-dipole interactions.

Concerning the pharmacological applications, coumarins were investigated as potential drugs in a wide array of pathologies, with a special mention to neurodegenerative diseases and cancer. Indeed, coumarins have shown very promising activity in the treatment of neurological disorders, like AD and PD, for their ability to selectively target enzymes involved in neurotransmitter metabolisms, such as AChE and MAO-B, at nanomolar concentrations. It is noteworthy that some of these derivatives such as **176** or **177** can cross the BBB maintaining a good hydrophilic/lipophilic balance which is essential to obtain a drug-like molecule. Furthermore, coumarins modulate many biological targets implicated in cancer, displaying antiproliferative activity in various cancer cell lines without affecting normal cells. Importantly, *in vivo* antitumoral effects were also observed for some derivatives like **211** or **213**. Since coumarins have favorable photophysical characteristics, they can be also utilized as photosensitizers in PDT which is gaining a growing attention in the recent years as minimally invasive therapy for the treatment of cancer.

It is worth to note that the coumarin scaffold is a valuable template for the development of MTDLs. Indeed, the concept “one drug one disease” has been overcome as several pathologies, such as cancer and neurodegenerative diseases, involve multifactorial events and, therefore, the modulation of a single target often does not result in adequate efficacy. Coumarins have been successfully used in this sense especially in the search of MTDLs for AD (compounds **176** and **177**) and therapeutics for tumors (compounds **208** and **209**).

Besides their activity on neurodegenerative diseases and cancer, coumarins can also be engineered towards antimicrobial, antiparasitic, antidiabetic, anti-inflammatory, skin-lightening, and antioxidant activities. For examples, coumarins carrying a thiazole or thiazolidinones are potential antityrosinase and antidiabetic drugs as well as excellent antibacterial agents characterized by less tendency to resistance respect to some antibacterials currently used in therapy. However, coumarin-isothiazole hybrids showed good antifungal effects, while coumarins carrying a quinazoline moiety are endowed with promising antiviral properties.

Despite the satisfactory results achieved, more investigations are needed to assess the metabolic stability and toxicity of coumarins. In animal-based studies, coumarins resulted to be carcinogenic at very high doses and the carcinogenic mechanism appears to be metabolism-mediated (Lake, 1999). However, these considerations are true for natural coumarins and cannot be applied to synthetic derivatives whose toxicity and metabolic pathways strongly rely on the substitution pattern, and therefore, require additional investigations.
